# Investigating vaccine-induced immunity and its effect in mitigating SARS-CoV-2 epidemics in China

**DOI:** 10.1186/s12916-022-02243-1

**Published:** 2022-01-31

**Authors:** Hengcong Liu, Juanjuan Zhang, Jun Cai, Xiaowei Deng, Cheng Peng, Xinghui Chen, Juan Yang, Qianhui Wu, Xinhua Chen, Zhiyuan Chen, Wen Zheng, Cécile Viboud, Wenhong Zhang, Marco Ajelli, Hongjie Yu

**Affiliations:** 1 School of Public Health, Fudan University, Key Laboratory of Public Health Safety, Ministry of Education, Shanghai, China; 2 Department of Infectious Diseases, Huashan Hospital, Fudan University, Shanghai, China; 3 Shanghai Institute of Infectious Disease and Biosecurity, Fudan University, Shanghai, China; 4 Division of International Epidemiology and Population Studies, Fogarty International Center, National Institutes of Health, Bethesda, MD USA; 5 Laboratory for Computational Epidemiology and Public Health, Department of Epidemiology and Biostatistics, Indiana University School of Public Health, Bloomington, IN USA

**Keywords:** COVID-19, Herd immunity, Vaccination program, Delta variant, SLIR model

## Abstract

**Background:**

To allow a return to a pre-COVID-19 lifestyle, virtually every country has initiated a vaccination program to mitigate severe disease burden and control transmission. However, it remains to be seen whether herd immunity will be within reach of these programs.

**Methods:**

We developed a compartmental model of SARS-CoV-2 transmission for China, a population with low prior immunity from natural infection. Two vaccination programs were tested and model-based estimates of the immunity level in the population were provided.

**Results:**

We found that it is unlikely to reach herd immunity for the Delta variant given the relatively low efficacy of the vaccines used in China throughout 2021 and the lack of prior natural immunity. We estimated that, assuming a vaccine efficacy of 90% against the infection, vaccine-induced herd immunity would require a coverage of 93% or higher of the Chinese population. However, even when vaccine-induced herd immunity is not reached, we estimated that vaccination programs can reduce SARS-CoV-2 infections by 50–62% in case of an all-or-nothing vaccine model and an epidemic starts to unfold on December 1, 2021.

**Conclusions:**

Efforts should be taken to increase population’s confidence and willingness to be vaccinated and to develop highly efficacious vaccines for a wide age range.

**Supplementary Information:**

The online version contains supplementary material available at 10.1186/s12916-022-02243-1.

## Background

The first-wave of novel coronavirus disease 2019 (COVID-19) in China subsided quickly after the implementation of strict containment measures and travel restrictions starting in March 2020 [1–4]. As of November 12, 2021, the COVID-19 pandemic has caused over 251 million reported cases and 5 million deaths globally [5]. The pandemic is far from over, as severe acute respiratory syndrome coronavirus 2 (SARS-CoV-2) has undergone some significant mutations and a number of variants have become widespread due to increased transmissibility and/or immune escape characteristics—e.g., variants Alpha [6–12], Beta [13, 14], Gamma [13, 15], and Delta [16–18]. Throughout the globe, a rapid surge of Delta variant cases suggests a clear competitive advantage compared with Alpha, Beta, and Gamma [16]; more than 90% of daily sequences from global initiative on sharing all influenza data (GISAID) are ascribable to the Delta variant since July 2021 [19]. Despite of no major epidemics, China has been experiencing several minor local outbreaks caused by imported cases of Delta variant, including the outbreaks in Guangzhou, Nanjing, and Zhengzhou city [20–22]. To suppress transmission, a large share of the world needs to have immunity to SARS-CoV-2, especially to the Delta variant.

Effective vaccines against COVID-19 represent the most viable option to suppress SARS-CoV-2 transmission globally. The effectiveness of vaccination programs depends on several key factors, including vaccine supply, willingness to receive the vaccine, vaccine efficacy, and the age groups targeted by the vaccination effort. However, current vaccination programs are all based on vaccines developed against the original SARS-CoV-2 lineage, and the efficacy seems be reduced against the Delta variant [23]. In China, home of about 1.4 billion people (~18% of the world population), 2.37 billion doses have been administered as of November 12, 2021 [24]; 76.5% of the whole population has been vaccinated with two doses, corresponding to 82.4% of the target population (i.e., individuals aged 3 years and older). However, it remains to be seen if the vaccine coverage may reach a level sufficient to achieve herd immunity. Countries around the globe are facing the same question.

The classical herd immunity level is defined as 1-1/*R*_*0*_, where *R*_*0*_ is the basic reproduction number—the average number of infections generated by a typical infectious individual in a fully susceptible population [25]. For a vaccine with efficacy VE that gives life-long protection, the level of herd immunity required to stop transmission is (1-1/*R*_*0*_)/VE. However, this estimate is an oversimplification of a complex phenomenon as it ignores the heterogeneities of actual human population (e.g., social mixing patterns, age-specific susceptibility to infection) [25, 26] as well as of vaccination (e.g., lifelong immunity, sterilizing vaccine). To overcome this limitation, here we integrate contact survey specific of the Chinese population [27] as well as official demographic statistics to develop an age-structured stochastic model to simulate SARS-CoV-2 transmission (Additional file 1: Fig. S1). We then use this model to evaluate whether herd immunity is achievable against the Delta variant or not via mass vaccination.

## Methods

### SARS-CoV-2 transmission and vaccination model

We built a compartmental model of SARS-CoV-2 transmission and vaccination, based on an age-structured stochastic susceptible-latent-infectious-removed (SLIR) scheme, accounting for heterogeneous contact patterns by age [27] and heterogeneous susceptibility to infection by age as estimated using contact tracing data in Hunan province of China [28]. In the model, the population is divided into four epidemiological categories: susceptible, latent, infectious, and removed, stratified by 16 age groups. Susceptible individuals can become infected after contact with an infectious individual according to the age-specific force of infection. The rate at which contacts occur is determined by the mixing patterns of each age group. The latent period and average generation time were set to be 4.4 [28–30] and 7 [31] days, respectively. We consider a basic reproductive number (*R*_*0*_) of 6.0 according to estimates for the SARS-CoV-2 Delta variant [1–4, 6–12, 16–18]. Simulations are initiated with 40 infectious individuals [32], corresponding to the number of cases first detected in a local outbreak in Beijing on June 11, 2020.

We consider a 2-dose vaccine that only susceptible individuals are eligible for vaccination (we recall that natural immunity is close to 0 in China as of November 2021 [33]) and that the duration of vaccine-induced immunity lasts longer than the time horizon considered in this study (i.e., 1 year). Details about the model and parameters are reported in Additional file 1: Sec. 1 and Tab. S1.

### Baseline scenario

As the baseline scenario, we considered the following assumptions:

i) **Epidemic seeding:** An epidemic is assumed to be triggered by 40 SARS-CoV-2 infectious individuals on December 1, 2021 [32].
ii) **Vaccination strategy:** Vaccines have been rolling out in China since November 30, 2020 [24], which is the earliest date reported by the government and have been extended to individuals aged 3+ years since early November, and we test two different vaccination strategies:
  - Strategy 1—random distribution of vaccines to individuals aged 12+ years, then extended to individuals aged 3+ years starting from November 1, 2021;
  - Strategy 2—random distribution of vaccines to individuals aged 3+ years since the start of the vaccination program, namely November 30, 2020.
  We considered that a fraction of the population (about 2%—Additional file 1: Tab. S2) is not eligible to receive the vaccine as pregnant women and individuals with allergies or other conditions are excluded from vaccination campaign in China as of November 2021 [34–40] (see Additional file 1: Sec. 2 for detail).
iii) **Vaccine capacity:** We used the historical data on daily administrated doses in China until November 2, 2021 (Additional file 1: Fig. S2), then projected the future daily capacity based on the average daily doses administrated over the period October 2–November 2, 2021 [24]. Thus, from November 3, 2021, and beyond, we estimated a daily vaccine administration capacity of 2.30 million doses for the China population (details are reported in Additional file 1: Sec. 3).
iv) **Vaccine efficacy:** The vaccine schedule requires two doses with 21-day interval. VE against infection for individuals aged 18–59 years old reaches the its maximum value 14 days after vaccinating 2 doses and is estimated at 54.3% for Delta variant [41–43]. This estimate is based on the efficacy measured against the original lineages and the reduction of neutralizing antibodies estimated for Delta variant in clinical studies (see Additional file 1: Tab. S1 for details). The relative VE against infection within 0–13 days after second dose comparted with maximum protection is 83.8% for Delta variant [44]. VE against death for individuals aged 18–59 years old is 93% for Delta variant [45–47]. We explored higher VE values against infection [48] and tested a two-dose schedule with a 14-day interval as sensitivity analyses (Additional file 1: Tab. S1). In addition, COVID-19 vaccines may not be equally effective across age groups in preventing infection. To understand the impact of this assumption, we also tested a relative VE of 50% and 75% for individuals aged 3–17 and 60+ years as compared to VE for individuals aged 18–59 years.
v) **Vaccine action:** We considered two mechanisms to model vaccine efficacy: an “all-or-nothing” vaccine (baseline analysis), in which the vaccine provides full protection to a fraction VE of individuals who are vaccinated and no protection to the remaining 1-VE vaccinated individuals. The second option we considered is a “leaky” vaccine in which all vaccinated individuals have a certain level of protection to the infection corresponding to VE [49].
vi) **Initial immunity:** As of November 2021, there is essentially no population immunity from natural infection in China [33]. For the sake of generalizability of the results to other countries that had widespread transmission, we explored a scenario where 30% of the population is initially immune to the infection, and the fraction of immune individuals by age group is proportional to the population size.
vii) **Susceptibility to infection by age:** Children under 15 years of age are estimated to have a lower susceptibility to SARS-CoV-2 infection as compared to adults (i.e., individuals aged 15 to 64 years), while individuals aged 65+ years have the highest susceptibility to infection [28].
viii) **Immunity duration:** We let the transmission model run for 1 year, assuming a life-long protection from natural infection or vaccination.
ix) **Disease burden:** The infection fatality ratio for original lineages manifest in 0.0923% for individuals aged 0–19, rising to 6.7959% for individuals aged over 80 years [50, 51]. The risk of death associated with the Delta variant compared to original lineages is 2.37 [52].

Comprehensive sensitivity analyses to evaluate the impact of the baseline assumptions on our results are carried out as well (Additional file 1: Tab. S1).

### Alternative vaccination scenarios

We tested three alternative scenarios to explore the potential for vaccination-induced herd immunity, where (i) the start of the epidemic is delayed from December 1, 2021, to January 1, 2022, and February 1, 2022; (ii) the value of the reproduction number in a fully susceptible population and under a certain level of non-pharmaceutical interventions (NPIs), denoted as $R^{NPIs}_{0}$ , varies between 1.1 and 6; (iii) combinations of scenarios i and ii. For scenario (ii), we did not explicitly model single NPIs such as case isolation, contact tracing, wearing masks, social distancing, and improved hygiene. Instead, the synergetic effect of these measures was considered as a reduction of the reproduction number.

### Data analysis

For each scenario, 100 stochastic simulations were performed, and mean and 95% confidence interval (95% CI) were then estimated.

We used the next-generation matrix (NGM) [53] approach to estimate the effective reproduction number, *R*_*e*_. Herd immunity is considered as achievable when *R*_*e*_ <1. Details are reported in Additional file 1: Sec. 4 and 5.

## Results

### Baseline scenario

**Fig. 1:**
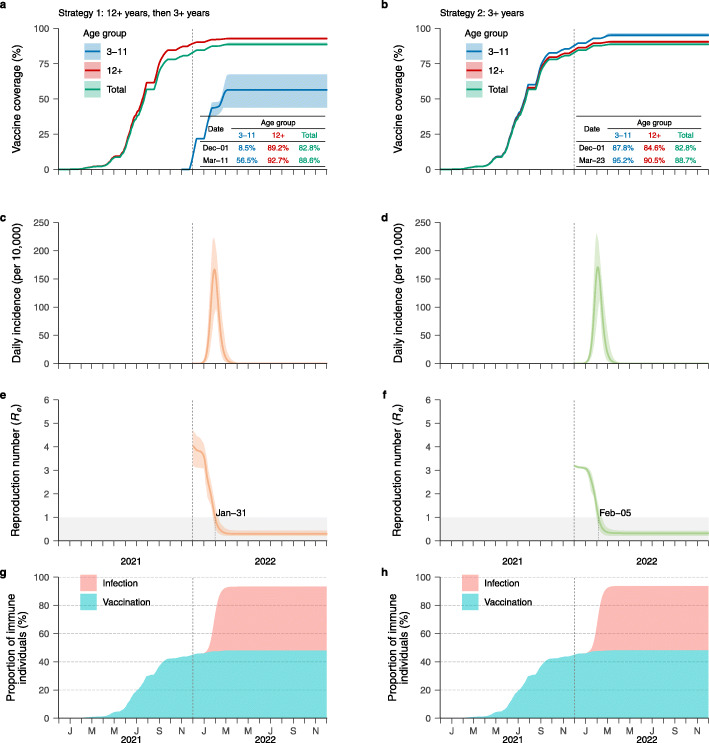
Time series of vaccine coverage, daily incidence, effective reproductive number, and proportion of immune individuals. **a** Age-specific vaccine coverage over time for strategy 1. The dotted lines correspond the start of epidemic. The inserted table shows the age-specific coverage for the two key time points (the start of epidemic (i.e., December 1, 2021) and the time that the coverage keeps constant (i.e., March 11)). The line corresponds to the mean value, while the shaded area represents 95% CI. **b** As **a**, but for strategy 2. **c** Daily incidence per 10,000 for strategy 1 (mean and 95% CI). **d** As **c**, but for strategy 2. **e** Effective reproduction number *R*_*e*_ over time (mean and 95% CI) for strategy 1. The shaded area in gray indicates the epidemic threshold *R*_*e*_ =1. The numbers around the shaded area indicate when *R*_*e*_ cross this threshold (i.e., January 31) for strategy 1. **f** As **e**, but for strategy 2. **g** Proportion of immune individuals due to either natural infection or vaccination over time for strategy 1. **h** As **g**, but for strategy 2

By forward simulating 1 year of epidemic and assuming no vaccine hesitancy, continued vaccination efforts would lead to a final coverage of 90.7% of the target population, which corresponds to 88.6% of the total population for strategy 1 (Fig. 1a). For strategy 2, the estimated coverage of the total population is 88.7% (Fig. 1b). Under any scenario, the mean daily incidence never reaches 250 over 10,000 residents (Fig. 1c, d). We estimated that the effective reproduction number at the time the infection is seeded (*R*_*e*_) is still well above the epidemic threshold, namely 4.03 (95% CI 3.19–4.70) and 3.18 (95% CI 3.15–3.24) for strategy 1 and 2, respectively (Fig. 1e, f). These estimates suggest that the vaccine coverage on December 1, 2021, is not enough to prevent onward transmission, regardless of the vaccination strategy. *R*_*e*_ is estimated to cross the epidemic threshold (i.e., 1) on January 31 and February 5, 2022, for strategy 1 and 2, respectively, due to the accumulation of immune individuals both through continued vaccination efforts and natural infections (Fig. 1g, h). The estimated infection attack rates (which includes all SARS-CoV-2 infections, independently of whether an individual develops symptoms or not) are 45.2% (95% CI 37.2–48.5%) and 45.7% (95% CI 40.6–48.8%) for strategies 1 and 2, respectively (Fig. 1g, h). Note that the proportion of vaccine-immune individuals stops increasing while the proportion of naturally immune individuals is still increasing. In fact, when all individuals are either vaccinated or infected, the proportion of vaccine-immune individuals will stop increasing. However, in this situation, the unprotected/partially protected vaccinated individuals can still be infected, which leads to an increase in the proportion of naturally immune individuals.

**Fig. 2:**
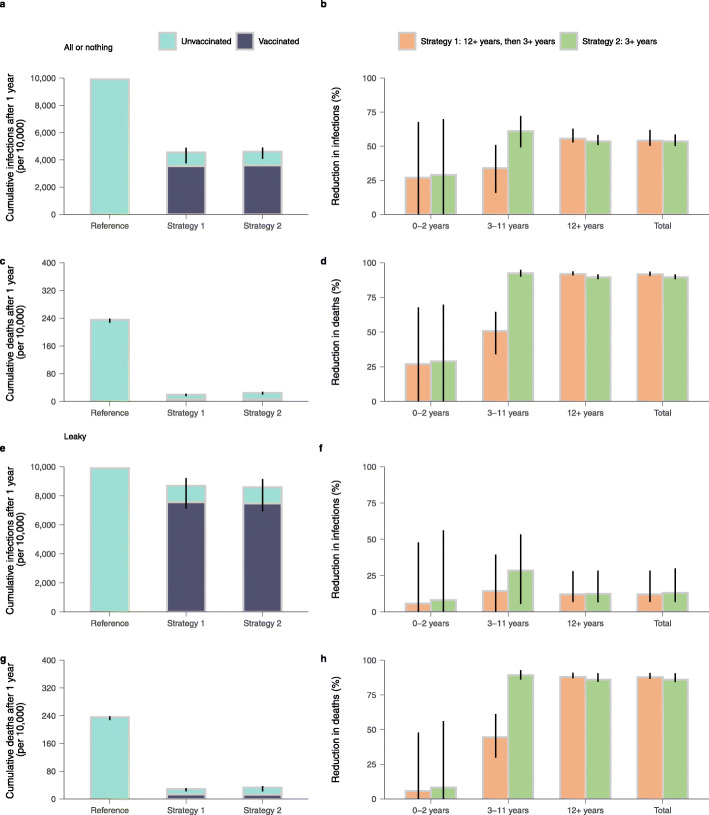
Disease burdens of COVID-19 in the baseline scenario. **a** Cumulative number of infections per 10,000 individuals after 1 simulated year for *reference scenario* and two vaccination strategies using “all-or-nothing” vaccine model (mean and 95% CI). **b** Reduction in infections (mean and 95% CI) with respect to the *reference scenario* in different age groups and the total population. The 95% CI of the reduction may cross 0 as the burden between reference scenario and vaccination scenario is approximately the same in some simulations. We thus trimmed the lower limit of 95% CI at 0 through the manuscript. **c**, **d** as for **a**, **b**, but for death. **e**–**h** as for **a**–**d**, but for “leaky” vaccine model

Although vaccine-induced immunity is not enough to prevent viral circulation, all the scenarios considered are associated with substantial mitigation of COVID-19 burden. We estimate the infection attack rates for the two vaccination strategies to decrease by more than 50% with respect to a reference scenario with no interventions (Fig. 2a, b). Both strategies lead to more than 90% reduction in the number of deaths (Fig. 2c, d). These results were based on the assumption of an “all-or-nothing” vaccine. To test the robustness of our findings to this assumption, we tested a “leaky” vaccine. In this case, we estimated a lower reduction of the infection attack rate (12% as compared to about 50%); however, we estimated a similar reduction in the number of deaths (about 85% as compared to about 90%), Fig. 2e–h.

The obtained results show that herd immunity cannot be reached by December 1, 2021, regardless of the adopted vaccination strategy when the *R*_*0*_ is set at 5 or 7 (Additional file 1: Fig. S3), when the initial number of seeds is varied in the range from 10 to 100 (Additional file 1: Fig. S4), and when equal susceptibility to infection by age is assumed (Additional file 1: Fig. S5). The same conclusion is obtained when we considered a more parsimonious model with 3 age groups (Additional file 1: Fig. S6). Finally, we also conducted a counterfactual analysis where we assume that a part of the population was already immune before the start of the vaccination campaign (similar to the situation in Western countries). Under this assumption, we found that a 30% initial immunity proportion would not lead to *R*_*e*_ below the epidemic threshold for two strategies before December 1, 2021, (Additional file 1: Fig. S7). As regards the parameters regulating the vaccination process, we found that the vaccine efficacy 14 days after second dose has the largest impact, followed by the vaccine efficacy of individuals aged 3–17 and 60+ relative to individuals aged 18–59 years (Additional file 1: Fig. S8 and S9). On the other hand, the relative vaccine efficacy within 0–13 days after second dose and the time interval between the first and second dose have a more moderate impact on the overall effectiveness of the analyzed vaccination strategies (Additional file 1: Fig. S10 and S11).

### Scenario 1: Delaying the start of the epidemic

**Fig. 3:**
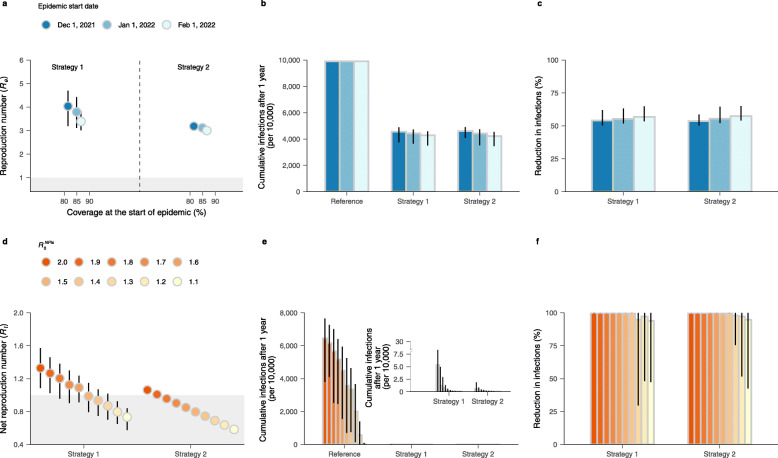
Impact of delaying the start of the epidemic and adopting NPIs. **a** Effective reproduction number *R*_*e*_ (mean and 95% CI) as a function of vaccine coverage at the time when infection is seeded. Colors refer to the scenario of delaying the start of the epidemic to different date. The shaded area in gray indicates *R*_*e*_ ≤1. **b** Cumulative number of infections per 10,000 individuals after 1 simulated year for *reference scenario* and two vaccination strategies (mean and 95% CI). **c** Reduction in infections (mean and 95% CI) with respect to the *reference scenario*. **d** As **a**, but for net reproduction number *R*_*t*_ (mean and 95% CI) adopting different intensity of NPIs. **e** As **b**, but for the scenario of adopting different intensity of NPIs. **f** As **c**, but for the scenario of adopting different intensity of NPIs

The findings presented thus far suggest that herd immunity against Delta variant cannot be built through vaccination by December 1, 2021. Next, we tested to what extent the start of a new epidemic wave needs to be delayed (e.g., by keeping strict restriction for international travels) to allow the immunity to build up in the population, potentially reaching herd immunity levels. According to the daily vaccine capacity used in the baseline scenario (based on the history of daily vaccination capacity data up to November 2, 2021), we estimated that *R*_*e*_ remains above the epidemic threshold for both two strategies even if the seeding of an epidemic is delayed to February 1, 2022 (Fig. 3a), while the reduction in infections increases to 56.8% and 57.4% for strategies 1–2, respectively. It is important to stress that the source of uncertainty in our estimates of *R*_*e*_ are the bootstrapped contact matrix by age and the posterior distribution of the susceptibility to infection by age. This explains why the estimated confidence interval of *R*_*e*_ for strategy 1 is wider that for strategy 2 (which, in Fig. 3a, is smaller than the size of the dot). In fact, for strategy 2, the vaccination is essentially uniform by age and thus the uncertainty on age-dependent parameters is negligible. On the contrary, for strategy 1, the young population is vaccinated at a later stage, which implies that the uncertainty on age-dependent parameters reflects in a larger uncertainty on *R*_*e*_. We also reported the impact of delaying the start of the epidemic on vaccine coverage and daily incidence in Additional file 1: Fig. S12.

### Scenario 2: Adopting NPIs in case of a new outbreak

The results presented so far suggest that herd immunity against Delta variant is not achievable at any time point. Adopting NPIs as a response to an epidemic outbreak can lower the transmission potential of the virus. It is thus worth investigating the synergetic effect of vaccination programs combined with NPIs of different intensity. It is important to note that we do not explicitly model every single measure to limit transmission (e.g., case isolation, contact tracing, wearing masks, social distancing, improved hygiene). These measures are implicit as concerted strategies that result in a decreased reproduction number. We explored $R^{NPIs}_{0}$ in the range 1.1–6.0 corresponding to different intensity of interventions. Values between 1 and 2 are showed in the main text, while larger values are shown in Additional file 1: Fig. S13. We also reported the impact of adopting NPIs in case of a new outbreak on daily incidence in Additional file 1: Fig. S14.

The mean net reproduction number (defined as the reproduction number accounting both for immunity and interventions) on December 1, 2021, for strategy 1 can be reduced to below 1 only when $R^{NPIs}_{0}$ ≤1.5, while for strategy 2, $R^{NPIs}_{0}$ can be up to 1.8 (Fig. 3d). By forward vaccinating and simulating 1 year of epidemic, substantial infections could be reduced (close to 100%) thanks to the synergetic effect of vaccination and NPIs (Fig. 3e, f). Note that the reductions in infections are obviously smaller than 100% for $R^{NPIs}_{0}$ ≤1.3, as the number of cumulative infections is extremely low in reference scenario.

### Scenario 3: Delaying the start of the epidemic and adopting NPIs

**Fig. 4:**
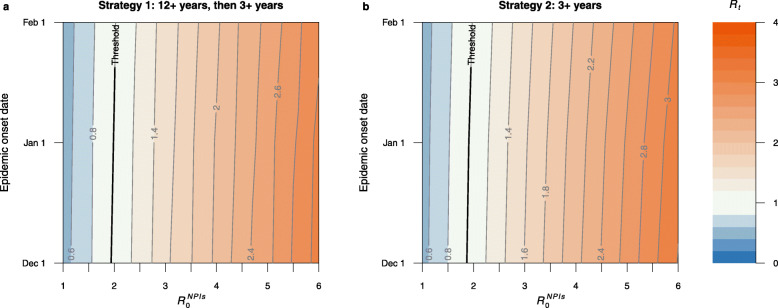
Impact of delaying the start of the epidemic start and adopting NPIs on net reproduction number. **a** Net reproduction number *R*_*t*_ as a function of $R^{NPIs}_{0}$ and epidemic start date for strategy 1. The bold line in black indicates *R*_*t*_ =1. **b** As **a**, but for strategy 2

To further improve the potential for vaccination-induced herd immunity and reduce COVID-19 burden, here we tested the combination of the two scenarios mentioned above: delaying the start of the epidemic and adopting NPIs of different level of intensity in response to a new outbreak. Should an epidemic start in December 2021–February 2022, strategies 1 and 2 can succeed in blocking transmission only if moderate NPIs ($R^{NPIs}_{0}$ in the range 1.5–2.0) are adopted (Fig. 4). The results of reduction in infections compared with reference scenario for two strategies are showed in Additional file 1: Fig. S15.

The effectiveness of age-targeted vaccination strategies depends on the age-mixing patterns of the population [54]. To test the robustness of our findings, we tested an alternative contact matrix for China [55] and found consistent results (Additional file 1: Fig. S16 and S17).

### Herd immunity threshold

Till now, herd immunity is unattainable for any vaccination strategy considering the relatively low efficacy (54.3%) of the analyzed vaccine in preventing the infection from the Delta. We thus explored the potential of herd immunity for the two vaccination strategies given a higher efficacy (95%) (Additional file 1: Fig. S18). We estimated that *R*_*e*_ can decrease below 1.0 for two strategies (Additional file 1: Fig. S18a). The estimated herd immunity thresholds under these two strategies are 91.3% and 84.5% respectively, which suggests that level of immunity needed to lead the effective reproduction number below the epidemic threshold is lower if vaccination is extended to individuals aged 3+ years early on.

We also estimated the infection attack rate under different vaccination coverages under the assumption that vaccination stops at the time the epidemic is seeded. This purely hypothetical scenario shows that when individuals aged 12+ years are prioritized (strategy 1), despite a fairly high estimated reproduction number when vaccine coverage equals to 80% (3.2), the estimated infection attack rate is relatively low (10.0%) (Additional file 1: Fig. S18b). In fact, given the age-targeted vaccination program and the lack of natural immunity, the susceptible population is mostly concentrated in the young population. The high number of contacts in younger age groups, combined with the high vaccination coverage in the rest of the population, lead to a fairly high reproduction number but, at the same time, the infections are focused on a small segment of the population only (young individuals) and thus the overall infection attack rate remains fairly low.

We also explored whether herd immunity is achievable or not and what is the herd immunity threshold by estimating *R*_*e*_ under the assumption that all individuals are eligible to be vaccinated and have vaccinated 2 doses before the epidemic starts. We considered vaccine efficacy in the range of 60–100% and explored different scenarios on vaccination coverage.

**Fig. 5:**
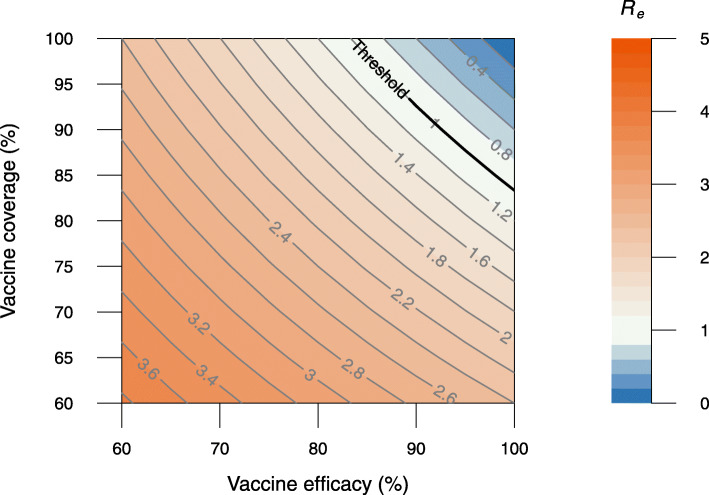
The impact of vaccine efficacy and vaccine coverage on the effective reproduction number. The bold line in black indicates the herd immunity threshold *R*_*e*_ =1

Our results show that, for a vaccine with an efficacy lower than 85%, herd immunity is unattainable, even in the extreme case where the vaccine coverage is 100% (Fig. 5). Vaccine-induced herd immunity may only be achievable with higher VE and coverage. For example, for a vaccine with 90% efficacy against infection from the Delta variant, more than 93% of the population would need to be vaccinated to reach herd immunity (Fig. 5). In the presence of NPIs, the net reproduction number can be reduced below the unit for lower vaccine efficacy and coverage values (Additional file 1: Fig. S19).

## Discussion

Our study evaluated the feasibility of reaching herd immunity against the SARS-CoV-2 Delta variant through vaccination, considering heterogeneity in population age structure, age-specific contact patterns, vaccine efficacy, and biological characteristics of SARS-CoV-2, including the basic reproduction number, susceptibility to infection by age, and key time-to-event periods (e.g., latent period, generation time). Our findings show that herd immunity is unlikely to be reached against the Delta variant given the relatively low efficacy of the current vaccines (developed against the original SARS-CoV-2 lineage), also in the presence of prior natural immunity up to 30%. Even considering vaccines with higher efficacy, our results show that extending the vaccination program to young children as soon plays a key role to increase the potential of reaching herd immunity and reduce the infection attack rate. If we consider a protection against the Delta variant of 90% (which goes beyond current vaccines), herd immunity would require the vaccination of 93% of the whole population. The adoption of NPIs could prevent the spread of a major epidemic wave even when the herd immunity level is not reached, but such an option obviously entails social and economic costs. Further, both strategies considered in this study would mitigate the overwhelming majority of infections.

Our study explored if and when vaccination-induced herd immunity can be reached in China. Under the hypotheses that the circulating strain has the same transmissibility as Delta variant and that the vaccination campaign will not slow down due to vaccine hesitancy, herd immunity seems to remain unreachable even in the extreme case where the vaccine coverage is 100%. Nonetheless, it is important to remark that the effectiveness of the vaccination program is impacted both by the natural immunity accumulated in the population (which is close to 0 in China as of November 2021) and the age structure of the population. In fact, in populations with a higher natural immunity level and a lower proportion of children, herd immunity may be achievable.

Our findings pointed to the importance of adopting NPIs and/or self-precautionary measures until herd immunity is reached or the burden of the epidemic becomes manageable. These measures can either help delay the seeding of the infection (e.g., strict border control measures) or should an epidemic start to unfold, mitigate its burden (e.g., social distancing, contact tracing, testing, wearing masks, hygiene practices, limiting contacts). However, questions remain about which NPIs need to be implemented, their intensity, and timing. Future studies are needed to address these questions.

A key role to determine the success of a vaccination campaign is played by the willingness-to-vaccine of the population. According to previous surveys on COVID-19 vaccine hesitancy, vaccine acceptance in China was estimated to vary between 60.4 and 91.3% for general population aged 18 years and above [56–59] and may be even lower for older adults [59]. Similar estimates were obtained for several other countries including the UK (71.5%) [60] and the USA (75.4%) [60]. Given these levels of vaccine hesitancy, achieving high levels of coverage may remain an elusive target. Efforts to increase population’s confidence and willingness to be vaccinated will thus be of paramount importance to allow a return to a pre-COVID-19 lifestyle. Our study shows that the spread of the more transmissible Delta variant has substantially increased the herd immunity threshold to a level that may not be feasible in any population, so that mitigation strategies become even more relevant.

Previous studies have estimated the herd immunity threshold either through natural infection or vaccination under the assumption of an homogenously mixed population [61–63], but heterogeneity in contact structure, age structure of the population, susceptibility to infection by age, and order in which individuals are vaccinated are all key factors shaping the herd immunity level [25]. To explore the impact of the heterogeneities included in the model on the obtained results, we tested an alternative model based on a fully homogeneous population, thus neglecting the contact structure, age structure of the population, susceptibility to infection by age, and order in which individuals are vaccinated that are accounted for in the main analysis (Additional file 1: Fig. S20). When considering *R*_*0*_=6.0 and vaccine efficacy against the infection = 95%, we estimated the theoretical herd immunity threshold (i.e., not accounting for waning of immunity and emergence of new variant with immune escape) to be 87.7% for the homogeneous model as compared to 91.3% and 84.5% of vaccination strategies 1 and 2 for the heterogeneous model. Our developed model is based on social mixing patterns estimated for the Shanghai population [27] and on China-specific data on COVID-19 epidemiology (population immunity, etc.). Nevertheless, the introduced modeling framework is flexible and can be tailored to other countries. We tested a scenario somehow resembling the situation in the USA, where we considered naturally immunity [64] and the adoption of BNT162b2/Pfizer vaccine, whose efficacy against the Delta variant was estimated at 79% [48]. Also, in this scenario, we estimated that herd immunity may not be reached (Additional file 1: Fig. S21). Moreover, vaccination hesitancy may jeopardize the vaccination effort in the USA and other Western countries as well.

This study is prone to the limitations pertaining to modeling exercises. First, VE against infections from the Delta variant was inferred instead of directly measures from epidemiological observations. Moreover, VE for children have not been estimated for the vaccines in use in China; therefore, we have assumed the same VE as in adults based on immunogenicity studies [65]. Given such a lack of field evidence, we have conducted a sensitivity analysis where a lower vaccine efficacy is assumed for children. The overall conclusions of the study do not change. Still, further data on age-specific vaccine efficacy could help refine the obtained estimates.

Second, we assumed that immunity induced either from infection or vaccination lasts more than the time horizon considered in the simulations (i.e., 1 year). There are both evidence from laboratory studies and the field suggesting that the protection lasts several months [66]. Despite these preliminary pieces of evidence, the duration of the immunity remains a research area of paramount importance and intrinsically linked to viral evolution. It is also possible that waning immunity will continue to provide protection against severe disease but only partial against infection or transmission, which affects the herd immunity threshold. Overall, the duration and quality of immunity will determine the periodicity of COVID-19 outbreaks globally [67, 68]. Moreover, booster vaccination may be an efficient way to improve the vaccine effectiveness [69–71]. For example, in Chile’s report about effectiveness of booster dose [69–71], the vaccine effectiveness of CoronaVac against infection increases from 50.18 to 70.89% after booster shot. The increased effectiveness of vaccination associated with the booster shot may contribute to increase immunity in the population and deserves further investigation.

Third, in the baseline scenario, we referred to an inactivated SARS-CoV-2 vaccine (BBIBP-CorV) taken to be 54.3% efficacious against the Delta variant infection. However, several other vaccines (including CoronaVac, WBIP-CorV, Ad5-nCoV, and ZF2001) are licensed and have been used in China. We varied vaccine efficacy up to 79% in sensitivity analyses. The main conclusion about the potential of herd immunity and the need to extend the vaccination campaign to children early as well as to use more efficacious vaccines is unaltered.

## Conclusion

In conclusion, based on the current evidence, reaching vaccine-induced herd immunity in a population with little/no natural immunity is challenging. A key step has been made on early November 2021 with the authorization of a vaccine for 3+ years old individuals. Minimize vaccine hesitancy in all age groups will be another key step to increase the immunity level of the population. These, together with highly efficacious vaccines or booster vaccinations, will be even more crucial given the possible emergence of new SARS-CoV-2 variants that are more transmissible or with immune escape. Importantly, even if herd immunity is unlikely to be reached due to waning of immunity and the emergence of new variants, vaccination will continue to dramatically reduce COVID-19 burden.

## Supplementary Information

**Additional file 1: Figure S1.** Schematic Figure of SARS-CoV-2 transmission and vaccination model. **Figure S2.** Vaccine administration capacity in China. **Figure S3.** Sensitivity analysis on the basic reproduction number. **Figure S4.** Sensitivity analysis on the initial number of infectious individuals. **Figure S5.** Sensitivity analysis on the susceptibility to infection. **Figure S6.** Sensitivity analysis on the number of age groups. **Figure S7.** Sensitivity analysis on the natural immunity. **Figure S8.** Sensitivity analysis on the maximum vaccine efficacy. **Figure S9.** Sensitivity analysis on the relative vaccine efficacy for individuals aged 3-17 and 60+ years relative to that of individuals aged 18-59 years. **Figure S10.** Sensitivity analysis on the vaccine efficacy within 14 days after second dose. **Figure S11.** Sensitivity analysis on the time intervals between the two doses. **Figure S12.** Impact of delaying the start of the epidemic on vaccine coverage and daily incidence. **Figure S13.** Impact of adopting NPIs in case of a new outbreak. **Figure S14.** Impact of adopting NPIs in case of a new outbreak on daily incidence. **Figure S15.** Impact of delaying the start of the epidemic and adopting NPIs on infections. **Figure S16.** Comparison of contact matrix in Shanghai and China. **Figure S17.** Impact of delaying the start of the epidemic start and adopting NPIs on estimated net reproduction number using China contact matrix. **Figure S18.** Effective reproduction number and infection attack rate under different vaccine coverage. **Figure S19.** Impact of vaccine efficacy and vaccine coverage on estimated net reproduction umber under different intensity of NPIs. **Figure S20.** Results of model with no age structure. **Figure S21.** Comparison between China and a scenario with natural immunity and an mRNA vaccine. **Tab S1.** Summary of parameters used to model Delta Strain. **Tab S2.** The proportion of pregnant women and vaccine contraindications by age groups.

## Data Availability

The code and data used to conduct these analyses are found at https://github.com/HengcongLiu/herd-immunity.

## Abbreviations

COVID-19: Coronavirus disease 2019
SARS-CoV-2: Severe acute respiratory syndrome coronavirus 2
GISAID: Global initiative on sharing all influenza data
SLIR: Susceptible-latent-infectious-removed
NPIs: Non-pharmaceutical interventions
95% CI: 95% confidence intervals
NGM: Next-generation matrix
