# Supplementary material for: Investigating vaccine-induced immunity and its effect in mitigating SARS-CoV-2 epidemics in China
Source: BMC Med. 2022 Jan 31;20:37. doi: 10.1186/s12916-022-02243-1 (PMC8801316; doi:10.1186/s12916-022-02243-1)
Supplement: Supplementary file 1 — Additional file 1: Figure S1. Schematic Figure of SARS-CoV-2 transmission and vaccination model. Figure S2. Vaccine administration capacity in China. Figure S3. Sensitivity analysis on the basic reproduction number. Figure S4. Sensitivity analysis on the initial number of infectious individuals. Figure S5. Sensitivity analysis on the susceptibility to infection. Figure S6. Sensitivity analysis on the number of age groups. Figure S7. Sensitivity analysis on the natural immunity. Figure S8. Sensitivity analysis on the maximum vaccine efficacy. Figure S9. Sensitivity analysis on the relative vaccine efficacy for individuals aged 3-17 and 60+ years relative to that of individuals aged 18-59 years. Figure S10. Sensitivity analysis on the vaccine efficacy within 14 days after second dose. Figure S11. Sensitivity analysis on the time intervals between the two doses. Figure S12. Impact of delaying the start of the epidemic on vaccine coverage and daily incidence. Figure S13. Impact of adopting NPIs in case of a new outbreak. Figure S14. Impact of adopting NPIs in case of a new outbreak on daily incidence. Figure S15. Impact of delaying the start of the epidemic and adopting NPIs on infections. Figure S16. Comparison of contact matrix in Shanghai and China. Figure S17. Impact of delaying the start of the epidemic start and adopting NPIs on estimated net reproduction number using China contact matrix. Figure S18. Effective reproduction number and infection attack rate under different vaccine coverage. Figure S19. Impact of vaccine efficacy and vaccine coverage on estimated net reproduction umber under different intensity of NPIs. Figure S20. Results of model with no age structure. Figure S21. Comparison between China and a scenario with natural immunity and an mRNA vaccine. Tab S1. Summary of parameters used to model Delta Strain. Tab S2. The proportion of pregnant women and vaccine contraindications by age groups. [file 12916_2022_2243_MOESM1_ESM.docx]

Supplementary Information for ：

**Investigating vaccine-induced immunity and its effect in mitigating SARS-CoV-2 epidemics in China**

Hengcong Liu^1^*, Juanjuan Zhang ^1,2,3^*, Jun Cai^1^, Xiaowei Deng^1^, Cheng Peng^1^, Xinghui Chen^1^, Juan Yang^1,2,3^, Qianhui Wu^1^, Xinhua Chen^1^, Zhiyuan Chen^1^, Wen Zheng^1^, Cécile Viboud^4^, Wenhong Zhang^2†^, Marco Ajelli^5†^ and Hongjie Yu^1,2,3†^

1. School of Public Health, Fudan University, Key Laboratory of Public Health Safety, Ministry of Education, Shanghai, China
2. Department of Infectious Diseases, Huashan Hospital, Fudan University, Shanghai, China
3. Shanghai Institute of Infectious Disease and Biosecurity, Fudan University, Shanghai, China
4. Division of International Epidemiology and Population Studies, Fogarty International Center, National Institutes of Health, Bethesda, MD, USA
5. Laboratory for Computational Epidemiology and Public Health, Department of Epidemiology and Biostatistics, Indiana University School of Public Health, Bloomington, IN, USA

*These authors contributed equally to this work.

^†^These authors are joint senior authors contributed equally to this work.

Corresponding authors: Hongjie Yu ([yhj@fudan.edu.cn](mailto:yhj@fudan.edu.cn))

1. **SARS-CoV-2 transmission and vaccination model**

We developed an age-stratified stochastic susceptible-latent-infectious-removed model of SARS-CoV-2 transmission. The population was divided into 16 age groups including 4 specific age groups (0-2, 3-4, 10-11, 12-14), 11 5-year age groups (5-9, 15-19, 20-24, …, 55-59, 60-64), and 1 age group for individuals aged 65 years and older. Age-mixing patterns were derived from a contact survey study conducted in Shanghai in 2017-2018 [27]. The model was used to simulate a set of vaccination programs.
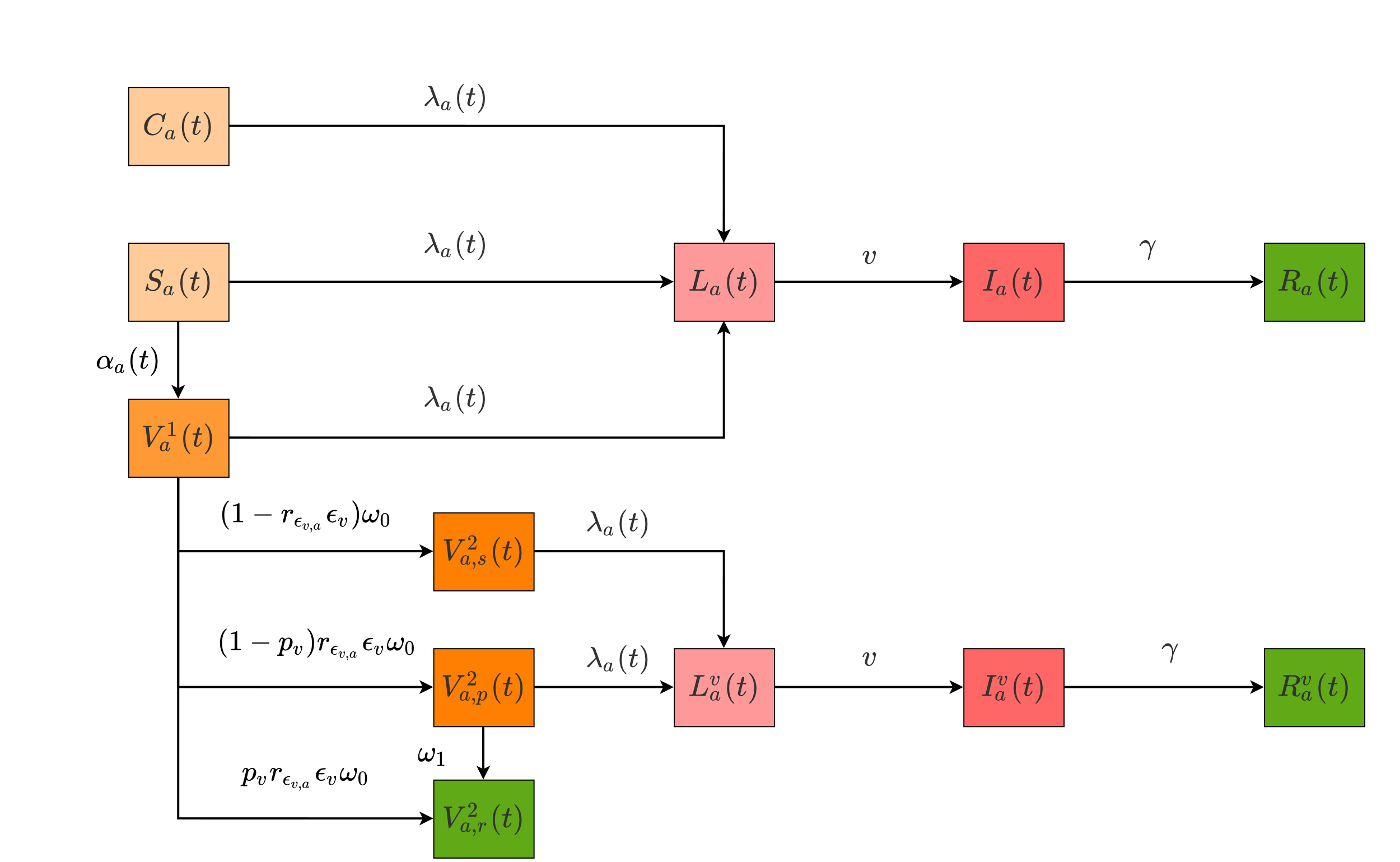


Fig. S1 Schematic figure of SARS-CoV-2 transmission and vaccination model.

The SARS-CoV-2 transmission and vaccination model is schematically represented in Fig. S1 and it is described by the following differential equations:

$\dot{C_{a}}=-\lambda_{a}C_{a}$ (1)

$\dot{S_{a}}=-\lambda_{a}S_{a}-\alpha_{a}S_{a}$ (2)

$\dot{L_{a}}=\lambda_{a}\left( C_{a}+S_{a}+V_{a}^{1} \right)-vL_{a}$ (3)

$\dot{I_{a}}=vL_{a}-\gamma I_{a}$ (4)

$\dot{R_{a}}=\gamma I_{a}$ (5)

$\dot{V_{a}^{1}}=\alpha_{a}S_{a}-\lambda_{a}V_{a}^{1}-\omega_{0}V_{a}^{1}$ (6)

$\dot{V_{a,s}^{2}}=\left( 1-{r_{\epsilon_{\upsilon,a}}\epsilon}_{\upsilon} \right)\omega_{0}V_{a}^{1}-\lambda_{a}V_{a,s}^{2}$ (7)

$\dot{V_{a,p}^{2}}=\left( 1-p_{v} \right){r_{\epsilon_{\upsilon,a}}\epsilon}_{\upsilon}\omega_{0}V_{a}^{1}-\lambda_{a}V_{a,p}^{2}-\omega_{1}V_{a,p}^{2}$ (8)

$\dot{V_{a,r}^{2}}=p_{v}{r_{\epsilon_{\upsilon,a}}\epsilon}_{\upsilon}\omega_{0}V_{a}^{1}+\omega_{1}V_{a,p}^{2}$ (9)

$\dot{L_{a}^{V}}=\lambda_{a}\left( V_{a,s}^{2}+V_{a,p}^{2} \right)-vL_{a}^{V}$ (10)

$\dot{I_{a}^{V}}=vL_{a}^{V}-\gamma I_{a}^{V}$ (11)

$\dot{R_{a}^{V}}=\gamma I_{a}^{V}$ (12)

where:

- $C_{a}$ represents the number of susceptible individuals with contraindications in age group $a$.
- $S_{a}$ represents the number of susceptible individuals in age group $a$.
- $L_{a}$ represents the number of latent individuals in age group $a$.
- $I_{a}$ represents the number of infectious individuals in age group $a$.
- $R_{a}$ represents the number of removed individuals in age group $a$.
- $V_{a}^{1}$ represents the number of individuals who have received first dose in age group $a$.
- $V_{a,s}^{2}$ represents the number of unprotected vaccinated individuals who have received second dose in age group $a$.
- $V_{a,p}^{2}$ represents the number of temporarily unprotected vaccinated individuals who have received second dose and will gain protection $1/{\omega_{1}}$ days later in age group $a$.
- $V_{a,r}^{2}$ represents the number of protected vaccinated individuals who have received second dose in age group $a$.
- $L_{a}^{V}$ represents the number of vaccinated latent individuals in age group $a$.
- $I_{a}^{V}$ represents the number of vaccinated infectious individuals in age group $a$.
- $R_{a}^{V}$ represents the number of vaccinated removed individuals in age group $a$.
- $\lambda_{a}$ represents the time- and age-dependent force of infection.
- $1/v$ corresponds to the mean latent period.
- $\gamma$ represents the recovery rate.
- $\alpha_{a}$ represents the probability of administration of first dose.
- $1/{\omega_{0}}$ represents time interval between administration of first and second dose.
- $1/{\omega_{1}}$ represents time needed to reach maximum vaccine efficacy after the administration of the second dose.
- $\epsilon_{\upsilon}$ represents maximum vaccine efficacy against infection for individuals aged 18-59 years.
- $p_{v}$ represents relative vaccine efficacy against infection right after administration of second dose as compared with maximum protection.
- $r_{\epsilon_{\upsilon,a}}$ represents relative vaccine efficacy of age group $a$ as compared with adults aged 18-59 years.

The time- and age-dependent force of infection $\lambda_{a}(t)$ to which susceptible individuals of age group $a$ are exposed to SARS-CoV-2 infection is defined as:

$\lambda_{a}(t)=\beta\sigma_{a}\sum_{\tilde{a}} C_{a, \tilde{a}}\frac{I_{\tilde{a}}+I_{\tilde{a}}^{V}}{N_{\tilde{a}}}$ (13)

Where:

- $\beta$ is a scaling factor shaping SARS-CoV-2 transmissibility.
- $\sigma_{a}$ is the susceptibility to SARS-CoV-2 infection for age group $a$.
- $C_{a,\tilde{a}}$ represents the age-group-specific contact matrix, whose elements describe the daily mean numbers of that a person in age group $a$ has with individuals in age group $\tilde{a}$.
- $N_{a}$ represents the number of individuals in age group $a$.

Considering vaccine administration capacity (see Additional file 1: Sec. 3), we prioritized those individuals who need to receive the second dose, and the remaining doses are randomly allocated to those who are eligible to receive the first dose. The number of doses given to each group is proportional to the population size. At each time $t$, the first dose of vaccination is administrated to a fraction of susceptible individuals in age group *a*:

$\alpha_{a}=\frac{d_{a}(t)}{S_{a}(t)}$ (14)

where $d_{a}(t)$ represents the number of first doses to be allocated to individuals in age group *a* at time $t$ under the designed vaccination strategies.

We modeled vaccine efficacy dependent on the delay between exposure and administration of the first and second dose. At the time of administration of the first dose, VE=0; then, it increases to $p_{v}\epsilon_{\upsilon}$ at the time of the second dose, and reaches the maximum protection $\epsilon_{\upsilon}$ after $1/{\omega_{1}}$ days.

We considered two mechanisms to model vaccine efficacy: “all-or-nothing” vaccine and “leaky” vaccine. Basically, for the “all-or-nothing” vaccine, individuals can be either: 1) successfully vaccinated, and in which case, they move to a vaccine protected compartment $V_{a,r}^{2}$ with a probability that depends on the vaccine efficacy at the various times (e.g., after the first dose, after the second dose); or 2) unsuccessfully vaccinated, in which case they have no protection (exactly as susceptible individuals), compartment $V_{a,s}^{2}$. For the “leaky” vaccine, all vaccinated individuals are partially protected and force of infection they are exposed to scaled by the vaccine efficacy.

A summary of model parameters is reported in Tab. S1.

**Tab. S1** Summary of parameters used to model Delta strain.

| Description | Baseline analysis | Sensitivity analysis |
| --- | --- | --- |
| Epidemiology | | |
| latent period | 4.4 days [28-30] | - |
| Generation time | 7.0 days [31] | - |
| Basic reproduction number ($\boldsymbol{R}_{\boldsymbol{0}}$) | 6.0 ^a^ [1-4, 6-12, 16-18] | 5.0, 7.0 |
| Susceptibility to infection by age ($\boldsymbol{\sigma}_{\boldsymbol{a}}$) | 0.58, 1 and 1.65 separately for 0-14, 15-64, and 65+ years [27] | Homogeneous susceptibility to infection |
| Initially infected individuals | 40 [32] | 10, 20, 100 |
| Initial natural immunity | 0 [33] | 10%, 20%, 30% |
| Epidemic starting date | December 1, 2021 | January 1, 2022,  February 1, 2022 |
| Vaccination | | |
| Interval between the administration of first and second dose ($\boldsymbol{1}/{\boldsymbol{\omega}_{\boldsymbol{0}}}$) | 21 days [42] | 14 days |
| Time needed to reach maximum protection after the administration of the second dose ($\boldsymbol{1}/{\boldsymbol{\omega}_{\boldsymbol{1}}}$) | 14 days [42] | - |
| Maximum vaccine efficacy in preventing the infection for individuals aged 18-59 years ($\boldsymbol{\epsilon}_{\boldsymbol{\upsilon}}$) | 54.3% ^b^ [41-43] | 0.6, 0.79 |
| Vaccine efficacy in preventing death for individuals aged 18-59 years | 93% [45-47] | - |
| Relative vaccine efficacy against infection right after administration of second dose as compared with maximum protection ($\boldsymbol{p}_{\boldsymbol{\upsilon}}$) | 83.8% ^c^ [44] | 0 |
| Relative vaccine efficacy for individuals aged 3-17 and 60+ years as compared to individuals aged 18-59 years ($\boldsymbol{r}_{\boldsymbol{\epsilon}_{\boldsymbol{v}}\boldsymbol{,a}}$) | 100% | 75%, 50% |
| Disease burden | | |
| Infection fatality ratio for the original lineages | 0.0923%, 0.1456%, 0.7259%, 3.7346%, and 6.7959% separately for 0-19, 20-39, 40-59, 60-79, and $\geq$ 80 years [50, 51] | - |
| Risk ratio of death associated with the Delta variant compared to the original lineages | 2.37 [52] | - |

^a^ $R_{0}$ of the original lineages is set to 2.5 [1-4] , and transmissibility of Alpha variant is 60% [6-12] higher as compared with that of original lineages, and transmissibility of Delta variant is 50% [16-18] higher as compared with that of Alpha variant. Thus, $R_{0}$ of Delta variant equals to 2.5*1.6*1.5=6.0.

^b^ Following the work by *Khoury et al* [43], we estimate vaccine protection against Delta variant infections by using two factors: i) the estimated efficacy against the infection from the original lineages for BBIBP-CorV vaccine [42], and ii) the reduction of neutralizing antibodies for the Delta variant estimated from *in vitro* neutralization assay [41]. Specifically, first, using the equations $E_{I} \left( n | n_{50},k \right)=\frac{1}{1+e^{-k(n-n_{50})}}$, we modelled the relationship between efficacy against the original lineage and neutralizing titers. Then, the distributions of changes in neutralizing antibodies for the Delta variants were added into the equation $P \left( n_{50},k, \mu_{s}, \sigma_{s} \right)=\int_{-\infty}^{+\infty} E_{I} \left( n | n_{50},k \right) f(n|\mu_{s},\sigma_{s})dn$ to estimate vaccine efficacy.

^c^ We assumed that individuals could be protected with a lower efficacy within 0-13 days after the second dose and the ratio is the same for both original lineages and Delta variant. In order to estimate the efficacy over time for BBIBP-Corv, we refer to another inactivated vaccine used in China (i.e., CoronaVac with 2 doses and 14 days apart). The efficacy for 0-13 days after second-dose and 14 days after second-dose are 42.5% and 50.7%, respectively [44]. Thus, the relative vaccine efficacy within 0-13 days after second-dose comparted with maximum protection is 42.5%/50.7% = 83.8%.

1. **Vaccine contraindication and pregnant women**

All individuals with vaccine contraindications and pregnant women are excluded from the target population of the simulated vaccination programs. Vaccine contraindications are defined according to the guideline reported by WHO [37-40]. The proportion of individuals with vaccine contraindications and pregnant women in Chinese population is listed in Tab. S2. We generated the proportion of individuals with at least 1 underlying condition of age group $a$, $P_{a}$, by using the following equation

$P_{a}=1-[\left( 1-p\left( C_{1} \right) \right)\left( 1-p\left( C_{2} \right) \right)\times\ldots\times\left( 1-p\left( C_{n} \right) \right)]$ (15)

Where $C_{n} (n=1, 2, 3, \ldots, 37)$ represents the underly conditions listed in Tab. S2 and $p(C_{n})$ represents the proportion of individuals with underlying condition $C_{n}$. We then used the ratio between real and estimated probability of individuals with at least 1 underlying condition reported in the literatures (68.2%) [34-36] to adjust probability of each group.

**Tab. S2** The proportion of pregnant women and vaccine contraindications by age groups.

| Proportion (%) | **Age group (years)** | | | | | | | | | | | | | | | | | |
| --- | --- | --- | --- | --- | --- | --- | --- | --- | --- | --- | --- | --- | --- | --- | --- | --- | --- | --- |
|  | 0-4 | 5-9 | 10-14 | 15-19 | 20-24 | 25-29 | 30-34 | 35-39 | 40-44 | 45-49 | 50-54 | 55-59 | 60-64 | 65-69 | 70-74 | 75-79 | 80-84 | 85+ |
| Pregnant women | 0.0 | 0.0 | 0.0 | 0.7 | 5.3 | 10.3 | 5.2 | 2.6 | 0.7 | 0.3 | 0.0 | 0.0 | 0.0 | 0.0 | 0.0 | 0.0 | 0.0 | 0.0 |
| Contraindications | 0.2 | 0.1 | 0.1 | 0.1 | 0.2 | 0.2 | 0.3 | 0.5 | 0.7 | 0.9 | 1.2 | 1.7 | 2.2 | 2.7 | 3.2 | 3.2 | 2.8 | 2.2 |
| Liver cancer due to NASH | 0.0 | 0.0 | 0.0 | 0.0 | 0.0 | 0.0 | 0.0 | 0.0 | 0.0 | 0.0 | 0.0 | 0.0 | 0.0 | 0.0 | 0.0 | 0.0 | 0.0 | 0.0 |
| Colon and rectum cancer | 0.0 | 0.0 | 0.0 | 0.0 | 0.0 | 0.0 | 0.1 | 0.1 | 0.1 | 0.2 | 0.3 | 0.4 | 0.6 | 0.8 | 1.0 | 1.0 | 0.9 | 0.7 |
| Lip and oral cavity cancer | 0.0 | 0.0 | 0.0 | 0.0 | 0.0 | 0.0 | 0.0 | 0.0 | 0.0 | 0.0 | 0.0 | 0.0 | 0.0 | 0.0 | 0.0 | 0.0 | 0.0 | 0.0 |
| Nasopharynx cancer | 0.0 | 0.0 | 0.0 | 0.0 | 0.0 | 0.0 | 0.0 | 0.1 | 0.1 | 0.1 | 0.1 | 0.1 | 0.1 | 0.1 | 0.1 | 0.0 | 0.0 | 0.1 |
| Other pharynx cancer | 0.0 | 0.0 | 0.0 | 0.0 | 0.0 | 0.0 | 0.0 | 0.0 | 0.0 | 0.0 | 0.0 | 0.0 | 0.0 | 0.0 | 0.0 | 0.0 | 0.0 | 0.0 |
| Gallbladder and biliary tract cancer | 0.0 | 0.0 | 0.0 | 0.0 | 0.0 | 0.0 | 0.0 | 0.0 | 0.0 | 0.0 | 0.0 | 0.0 | 0.0 | 0.0 | 0.0 | 0.0 | 0.0 | 0.0 |
| Pancreatic cancer | 0.0 | 0.0 | 0.0 | 0.0 | 0.0 | 0.0 | 0.0 | 0.0 | 0.0 | 0.0 | 0.0 | 0.0 | 0.0 | 0.0 | 0.0 | 0.0 | 0.0 | 0.0 |
| Malignant skin melanoma | 0.0 | 0.0 | 0.0 | 0.0 | 0.0 | 0.0 | 0.0 | 0.0 | 0.0 | 0.0 | 0.0 | 0.0 | 0.0 | 0.0 | 0.0 | 0.0 | 0.0 | 0.0 |
| Ovarian cancer | 0.0 | 0.0 | 0.0 | 0.0 | 0.0 | 0.0 | 0.0 | 0.0 | 0.0 | 0.0 | 0.0 | 0.0 | 0.0 | 0.0 | 0.0 | 0.0 | 0.0 | 0.0 |
| Testicular cancer | 0.0 | 0.0 | 0.0 | 0.0 | 0.0 | 0.0 | 0.0 | 0.0 | 0.0 | 0.0 | 0.0 | 0.0 | 0.0 | 0.0 | 0.0 | 0.0 | 0.0 | 0.0 |
| Kidney cancer | 0.0 | 0.0 | 0.0 | 0.0 | 0.0 | 0.0 | 0.0 | 0.0 | 0.0 | 0.0 | 0.0 | 0.0 | 0.0 | 0.0 | 0.1 | 0.0 | 0.0 | 0.0 |
| Bladder cancer | 0.0 | 0.0 | 0.0 | 0.0 | 0.0 | 0.0 | 0.0 | 0.0 | 0.0 | 0.0 | 0.0 | 0.1 | 0.1 | 0.1 | 0.2 | 0.2 | 0.1 | 0.1 |
| Brain and central nervous system cancer | 0.0 | 0.0 | 0.0 | 0.0 | 0.0 | 0.0 | 0.0 | 0.0 | 0.0 | 0.0 | 0.0 | 0.0 | 0.0 | 0.0 | 0.0 | 0.0 | 0.0 | 0.0 |
| Thyroid cancer | 0.0 | 0.0 | 0.0 | 0.0 | 0.0 | 0.0 | 0.0 | 0.0 | 0.0 | 0.0 | 0.0 | 0.0 | 0.0 | 0.0 | 0.0 | 0.0 | 0.0 | 0.0 |
| Mesothelioma | 0.0 | 0.0 | 0.0 | 0.0 | 0.0 | 0.0 | 0.0 | 0.0 | 0.0 | 0.0 | 0.0 | 0.0 | 0.0 | 0.0 | 0.0 | 0.0 | 0.0 | 0.0 |
| Larynx cancer | 0.0 | 0.0 | 0.0 | 0.0 | 0.0 | 0.0 | 0.0 | 0.0 | 0.0 | 0.0 | 0.0 | 0.0 | 0.1 | 0.1 | 0.1 | 0.1 | 0.1 | 0.0 |
| Tracheal, bronchus, and lung cancer | 0.0 | 0.0 | 0.0 | 0.0 | 0.0 | 0.0 | 0.0 | 0.0 | 0.0 | 0.0 | 0.1 | 0.1 | 0.2 | 0.3 | 0.4 | 0.4 | 0.4 | 0.3 |
| Breast cancer | 0.0 | 0.0 | 0.0 | 0.0 | 0.0 | 0.0 | 0.1 | 0.2 | 0.3 | 0.3 | 0.4 | 0.5 | 0.6 | 0.6 | 0.6 | 0.6 | 0.5 | 0.5 |
| Cervical cancer | 0.0 | 0.0 | 0.0 | 0.0 | 0.0 | 0.0 | 0.0 | 0.1 | 0.1 | 0.1 | 0.1 | 0.1 | 0.1 | 0.0 | 0.0 | 0.0 | 0.0 | 0.0 |
| Uterine cancer | 0.0 | 0.0 | 0.0 | 0.0 | 0.0 | 0.0 | 0.0 | 0.0 | 0.0 | 0.1 | 0.1 | 0.1 | 0.1 | 0.1 | 0.1 | 0.0 | 0.0 | 0.0 |
| Prostate cancer | 0.0 | 0.0 | 0.0 | 0.0 | 0.0 | 0.0 | 0.0 | 0.0 | 0.0 | 0.0 | 0.0 | 0.1 | 0.2 | 0.4 | 0.5 | 0.6 | 0.6 | 0.3 |
| Esophageal cancer | 0.0 | 0.0 | 0.0 | 0.0 | 0.0 | 0.0 | 0.0 | 0.0 | 0.0 | 0.0 | 0.0 | 0.1 | 0.1 | 0.1 | 0.2 | 0.2 | 0.2 | 0.1 |
| Stomach cancer | 0.0 | 0.0 | 0.0 | 0.0 | 0.0 | 0.0 | 0.0 | 0.0 | 0.1 | 0.1 | 0.1 | 0.2 | 0.3 | 0.3 | 0.4 | 0.4 | 0.4 | 0.2 |
| Liver cancer due to hepatitis B | 0.0 | 0.0 | 0.0 | 0.0 | 0.0 | 0.0 | 0.0 | 0.0 | 0.0 | 0.0 | 0.0 | 0.0 | 0.0 | 0.0 | 0.0 | 0.0 | 0.0 | 0.0 |
| Liver cancer due to hepatitis C | 0.0 | 0.0 | 0.0 | 0.0 | 0.0 | 0.0 | 0.0 | 0.0 | 0.0 | 0.0 | 0.0 | 0.0 | 0.0 | 0.0 | 0.0 | 0.0 | 0.0 | 0.0 |
| Liver cancer due to alcohol use | 0.0 | 0.0 | 0.0 | 0.0 | 0.0 | 0.0 | 0.0 | 0.0 | 0.0 | 0.0 | 0.0 | 0.0 | 0.0 | 0.0 | 0.0 | 0.0 | 0.0 | 0.0 |
| Liver cancer due to other causes | 0.0 | 0.0 | 0.0 | 0.0 | 0.0 | 0.0 | 0.0 | 0.0 | 0.0 | 0.0 | 0.0 | 0.0 | 0.0 | 0.0 | 0.0 | 0.0 | 0.0 | 0.0 |
| Myelodysplastic, myeloproliferative, and other hematopoietic neoplasms | 0.0 | 0.0 | 0.0 | 0.0 | 0.0 | 0.0 | 0.0 | 0.0 | 0.0 | 0.1 | 0.1 | 0.2 | 0.3 | 0.4 | 0.4 | 0.4 | 0.4 | 0.3 |
| Acute lymphoid leukemia | 0.1 | 0.0 | 0.0 | 0.0 | 0.0 | 0.0 | 0.0 | 0.0 | 0.0 | 0.0 | 0.0 | 0.0 | 0.0 | 0.0 | 0.1 | 0.0 | 0.0 | 0.0 |
| Chronic lymphoid leukemia | 0.0 | 0.0 | 0.0 | 0.0 | 0.0 | 0.0 | 0.0 | 0.0 | 0.0 | 0.0 | 0.0 | 0.0 | 0.0 | 0.0 | 0.0 | 0.0 | 0.0 | 0.0 |
| Acute myeloid leukemia | 0.0 | 0.0 | 0.0 | 0.0 | 0.0 | 0.0 | 0.0 | 0.0 | 0.0 | 0.0 | 0.0 | 0.0 | 0.0 | 0.0 | 0.0 | 0.0 | 0.0 | 0.0 |
| Chronic myeloid leukemia | 0.0 | 0.0 | 0.0 | 0.0 | 0.0 | 0.0 | 0.0 | 0.0 | 0.0 | 0.0 | 0.0 | 0.0 | 0.0 | 0.0 | 0.0 | 0.0 | 0.0 | 0.0 |
| Other leukemia | 0.1 | 0.1 | 0.0 | 0.0 | 0.0 | 0.0 | 0.0 | 0.0 | 0.0 | 0.0 | 0.0 | 0.0 | 0.0 | 0.0 | 0.0 | 0.0 | 0.0 | 0.0 |
| Hodgkin lymphoma | 0.0 | 0.0 | 0.0 | 0.0 | 0.0 | 0.0 | 0.0 | 0.0 | 0.0 | 0.0 | 0.0 | 0.0 | 0.0 | 0.0 | 0.0 | 0.0 | 0.0 | 0.0 |
| Non-Hodgkin lymphoma | 0.0 | 0.0 | 0.0 | 0.0 | 0.0 | 0.0 | 0.0 | 0.0 | 0.0 | 0.0 | 0.0 | 0.0 | 0.1 | 0.1 | 0.1 | 0.1 | 0.1 | 0.1 |
| Multiple myeloma | 0.0 | 0.0 | 0.0 | 0.0 | 0.0 | 0.0 | 0.0 | 0.0 | 0.0 | 0.0 | 0.0 | 0.0 | 0.0 | 0.0 | 0.0 | 0.0 | 0.0 | 0.0 |
| Other malignant neoplasms | 0.1 | 0.0 | 0.1 | 0.1 | 0.0 | 0.0 | 0.0 | 0.0 | 0.1 | 0.1 | 0.1 | 0.1 | 0.2 | 0.2 | 0.3 | 0.3 | 0.2 | 0.2 |

1. **Vaccine administration capacity**

We systematically collected information about COVID-19 vaccine administration in China from the press conferences of the Joint Prevention and Control Mechanism of the State Council [24] to Nov 2, 2021. The first data is recorded on November 30, 2020, and the vaccination program is targeting all individuals aged 12 years and older. Since the number of administrated doses is not released every day at the beginning, we calculated the mean administrated doses between 2 data points. We estimated the mean doses from October 2, 2021 to November 2, 2021 to estimate vaccine administration capacity from November 3, 2021 and beyond.


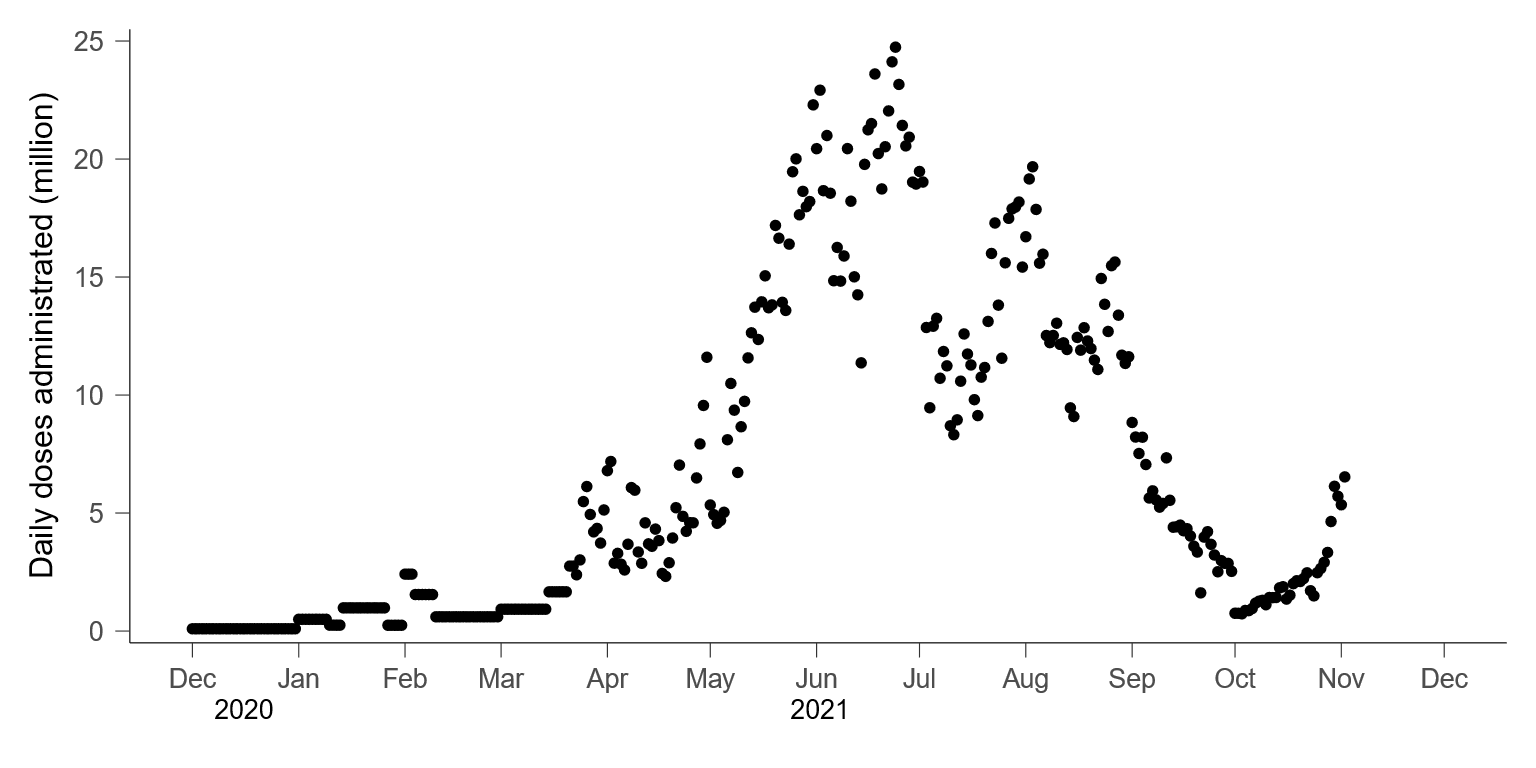


Fig. S2 Vaccine administration capacity in China.

1. **Transmission rate and basic reproduction number**

The basic reproduction number $R_{0}$ with age structure can be computed by using the Next-Generation matrix (NGM) approach [53] as:

$R_{0}=\frac{\beta}{\gamma}\rho(K_{1})$ (16)

Where $\rho(K_{1})$ presents the dominant eigenvalue of combined matrix $K_{1}$,

$K_{1}=\left[ \begin{matrix} \sigma_{1}C_{11}\frac{N_{1}}{N_{1}} & \cdots& \sigma_{1}C_{1n}\frac{N_{1}}{N_{n}} \\ \vdots& \ddots& \vdots\\ \sigma_{n}C_{n1}\frac{N_{n}}{N_{1}} & \cdots& \sigma_{n}C_{nn}\frac{N_{n}}{N_{n}} \end{matrix} \right]_{n}$ (17)

Thus, infectious rate $\beta$ can be computed based on equation from (15) as

$\beta=\frac{\gamma\times R_{0}}{\rho(K_{1})}$ (18)

1. **Estimation of the effective reproduction number**

The effective reproduction number $R_{e}$ accounts for the immunity in the population (either natural or from vaccination). It can be computed by using the Next-Generation matrix (NGM) approach as follows:

$R_{e}=\frac{\beta}{\gamma}\rho(K_{2})$ (19)

Where $\rho(K_{2})$ presents the dominant eigenvalue of combined matrix $K_{2}$,

$K_{2}=\left[ \begin{matrix} \sigma_{1}C_{11}\frac{N_{1}-N_{1}*c_{1}*\epsilon_{v}}{N_{1}} & \cdots& \sigma_{1}C_{1n}\frac{N_{1}-N_{1}*c_{1}*\epsilon_{v}}{N_{n}} \\ \vdots& \ddots& \vdots\\ \sigma_{n}C_{n1}\frac{N_{n}-N_{n}*c_{n}*\epsilon_{v}}{N_{1}} & \cdots& \sigma_{n}C_{nn}\frac{N_{n}-N_{n}*c_{n}*\epsilon_{v}}{N_{n}} \end{matrix} \right]_{n}$ (20)

Where $c_{a}$ represents the vaccine coverage of age group $a$ and $\epsilon_{v}$ represents the vaccine efficacy in preventing the infection.

1. **Sensitivity analyses**
   1. **Basic reproduction number**

In the main analysis, the basic reproduction number of the Delta variant was set to be 6.0. We performed a sensitivity analysis assuming $R_{0}$= 5.0 and 7.0. The estimated values of $R_{e}$ substantially increases with $R_{0}$. However, we estimated that the reduction of the cumulative infections decreases slightly while increasing $R_{0}$ (Fig. S3).


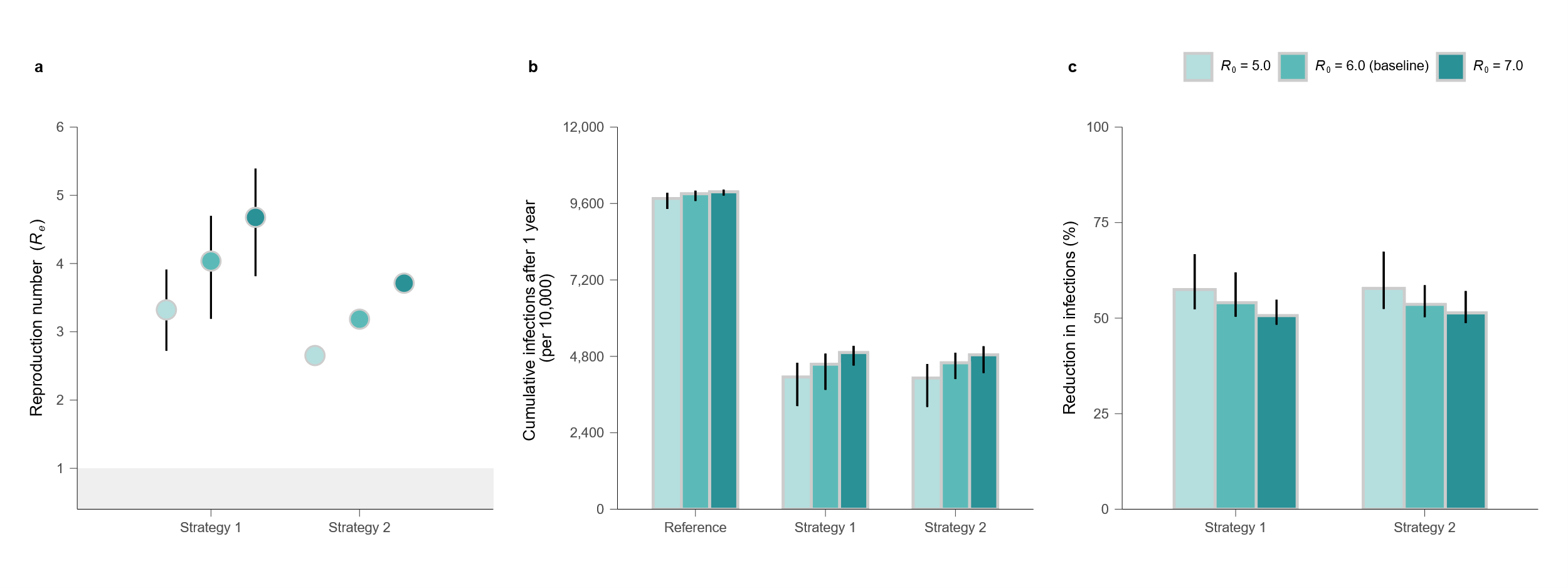


**Fig. S3 Sensitivity analysis on the basic reproduction number.** **a** Estimated effective reproduction number $R_{e}$ (mean and 95% CI) at the start of epidemic (December 1, 2021) for the two analyzed vaccination strategies. **b** Cumulative number of infections (mean and 95% CI) after 1 year for the *reference scenario* and two analyzed vaccination strategies. **c** Reduction in the cumulative number of infections (mean and 95% CI) due to vaccination with respect to the *reference scenario*.

- 1. **Initial number of infectious individuals**

In the main analysis, we used 40 infectious individuals to initialize the epidemic (Fig. 2). We performed a sensitivity analysis on the initial number of infectious individuals, namely 10, 20, and 100 infectious individuals. As we are looking a final outcome (and not to transient dynamics), all the obtained results are substantially independent of the initial number of infectious individuals (Fig. S4).


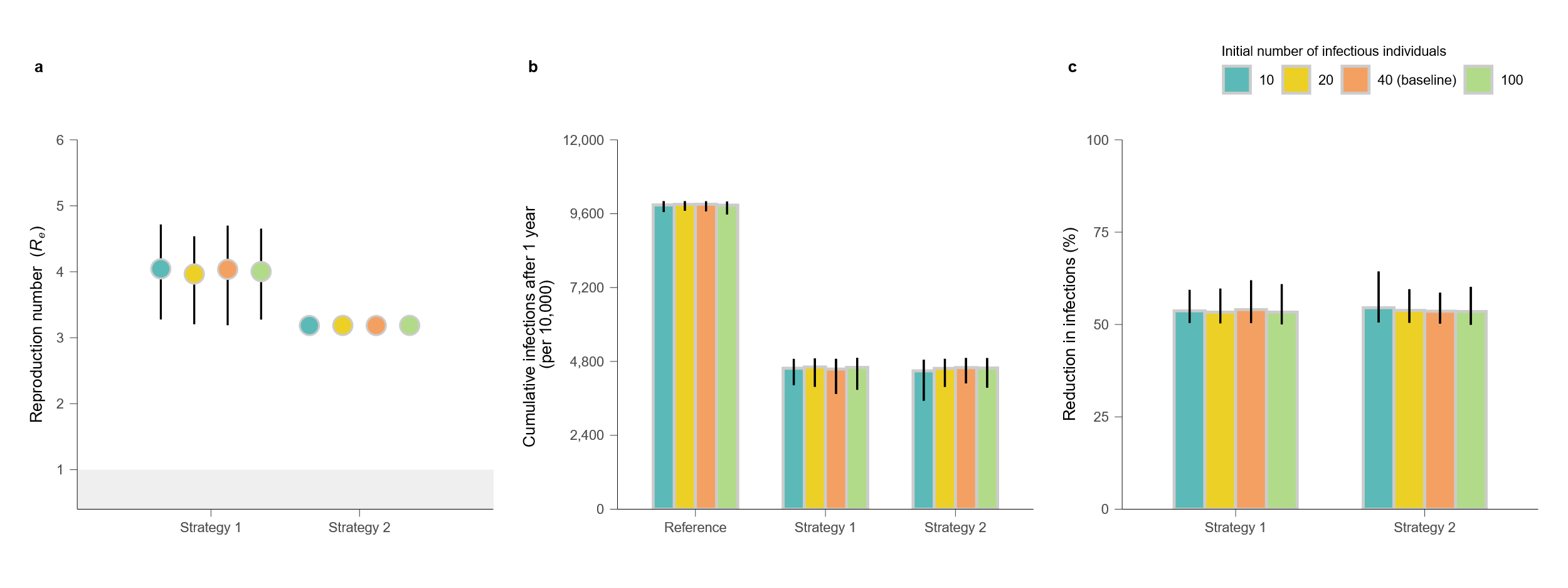


Fig. S4 Sensitivity analysis on the initial number of infectious individuals. a Estimated effective reproduction number $\boldsymbol{R}_{\boldsymbol{e}}$ (mean and 95% CI) at the start of epidemic (December 1, 2021) for two analyzed vaccination strategies. b Cumulative number of infections (mean and 95% CI) after 1 year for the *reference scenario* and two analyzed vaccination strategies. c Reduction in infections (mean and 95% CI) due to vaccination with respect to the *reference scenario*.

- 1. **Susceptibility to infection**

In the main analysis, we considered age-specific susceptibility to infection (Fig. 2). We performed a sensitivity analysis where susceptibility to infection is assumed to be homogeneous by age (Fig. S5). Slight differences are observed between the homogeneous and heterogeneous scenarios.


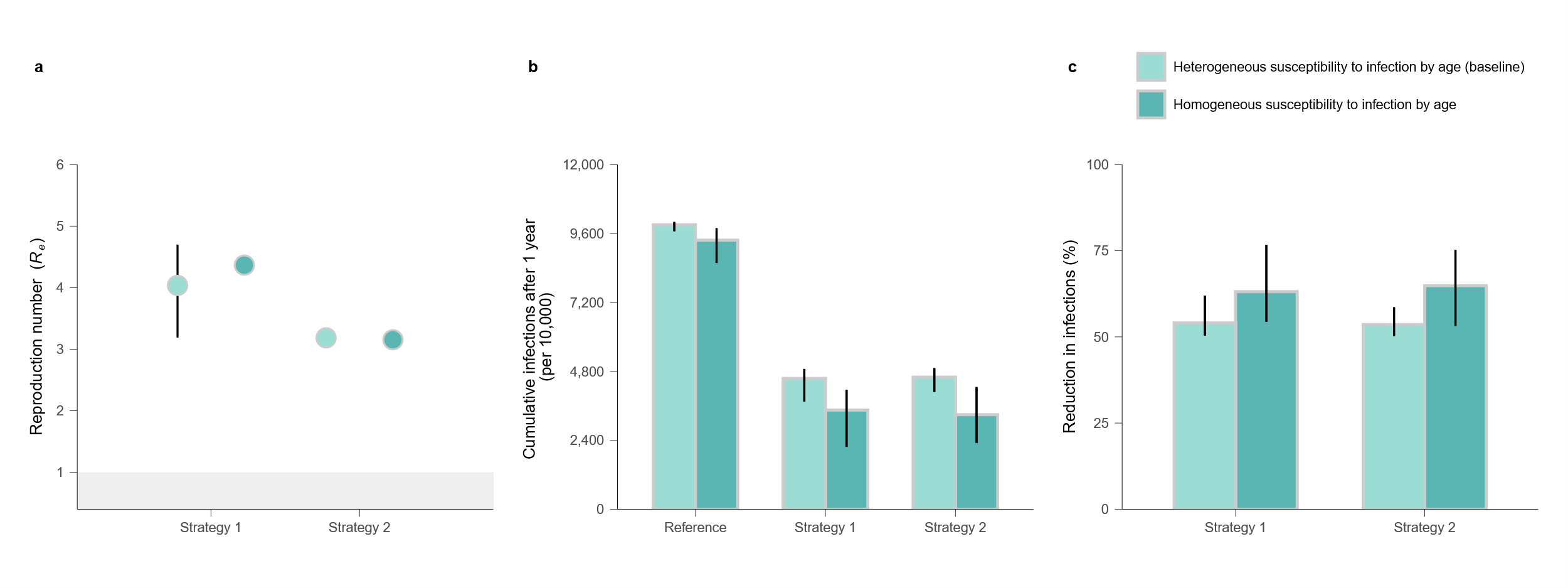


**Fig. S5 Sensitivity analysis on the susceptibility to infection.** **a** Estimated effective reproduction number $R_{e}$ (mean and 95% CI) at the start of epidemic (December 1, 2021) for the two analyzed vaccination strategies. **b** Cumulative number of infections (mean and 95% CI) after 1 year for the *reference scenario* and the two analyzed vaccination strategies. **c** Reduction in infections (mean and 95% CI) due to vaccination with respect to the *reference scenario*.

- 1. **Number of age groups**

In the main analysis, we considered 16 age groups (Fig. 2). We performed an analysis where three age groups are considered: 0-17, 18-59, and 60+ years (Fig. S6). The differences between results are visible, and we trusted the results of using 16 age groups.


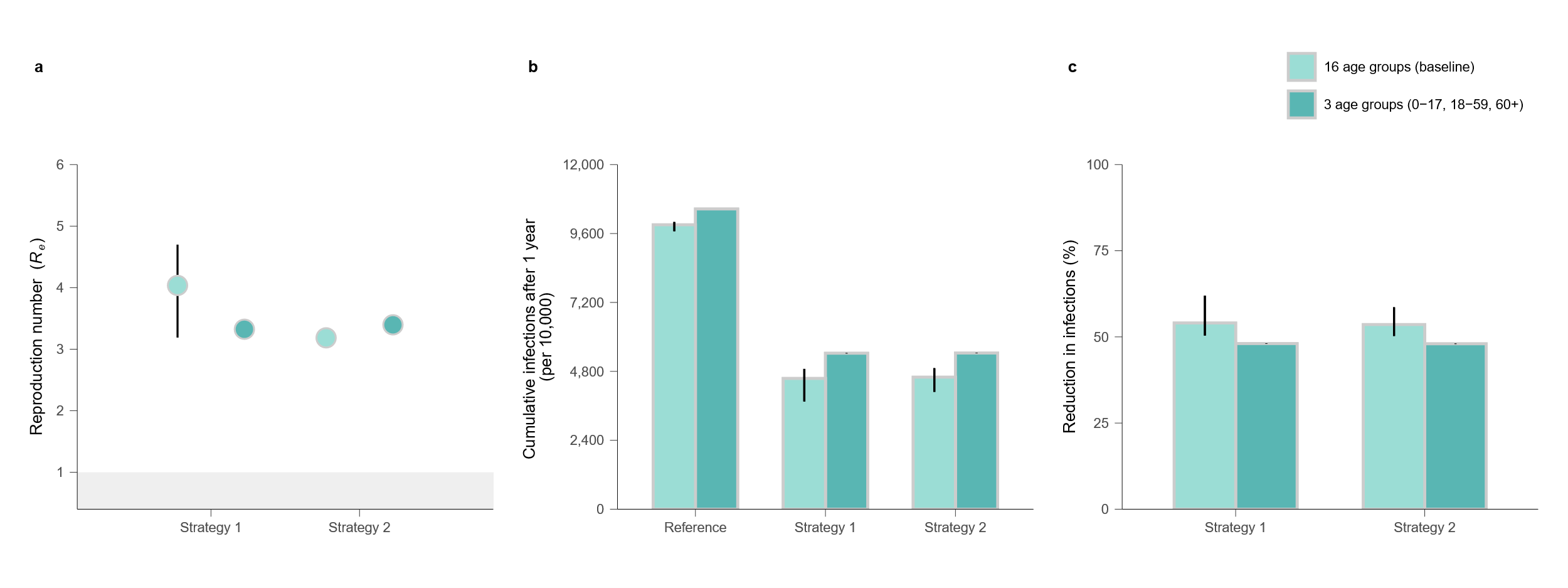


**Fig. S6 Sensitivity analysis on the number of age groups.** **a** Estimated effective reproduction number $R_{e}$ (mean and 95% CI) at the start of epidemic (December 1, 2021) for the two analyzed vaccination strategies. **b** Cumulative number of infections (mean and 95% CI) after 1 year for the *reference scenario* and the two analyzed vaccination strategies. **c** Reduction in infections (mean and 95% CI) due to vaccination with respect to the *reference scenario*.

- 1. **Natural immunity**

In the main analysis, we considered a population with no pre-existing natural immunity (Fig. 2). We performed a sensitivity analysis where the pre-existing natural immunity was set at 10%, 20%, and 30%. The estimated values of $R_{e}$ and cumulative infections after one year decrease with the increase of natural immunity, and the two vaccination strategies have a comparable mitigation effect (Fig. S7).


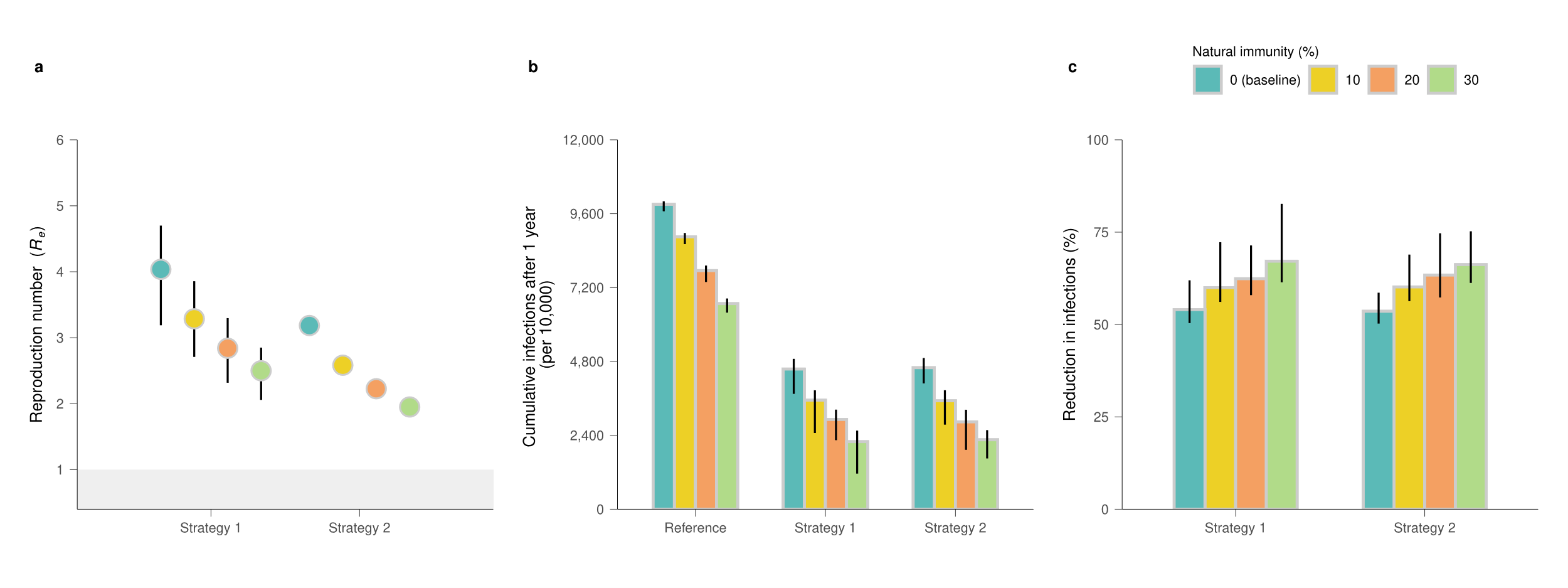


**Fig. S7 Sensitivity analysis on the natural immunity.** **a** Estimated effective reproduction number $R_{e}$ (mean and 95% CI) at the start of epidemic (December 1, 2021) for the two analyzed vaccination strategies. **b** Cumulative number of infections (mean and 95% CI) after 1 year for the *reference scenario* and the two analyzed vaccination strategies. **c** Reduction in infections (mean and 95% CI) due to vaccination with respect to the *reference scenario*.

- 1. **Vaccine efficacy**

In the main analysis, we considered a two-dose vaccine whose efficacy against SARS-CoV-2 infections was set to 54.3% [41-43] for all age groups (Fig. 2). We performed three sensitivity analysis on vaccine efficacy. In the first one, we varied the maximum vaccine efficacy up to 79%, in line with the vaccine efficacy for BioNTech/Pfizer [48] (Fig. S8); second, we varied the vaccine efficacy for individuals aged 3-17 and 60+ years relative to that of individuals aged 18-59 years (Fig. S9); third, we explored different vaccine efficacy within 14 days after the second dose (Fig. S10). We found that the reproduction number and cumulative infections after 1 year decrease with increase of maximum vaccine efficacy and relative vaccine efficacy for individuals age 3-17 and 60+ years, while the obtained results are similar when considering different vaccine efficacy within 14 days after second dose.


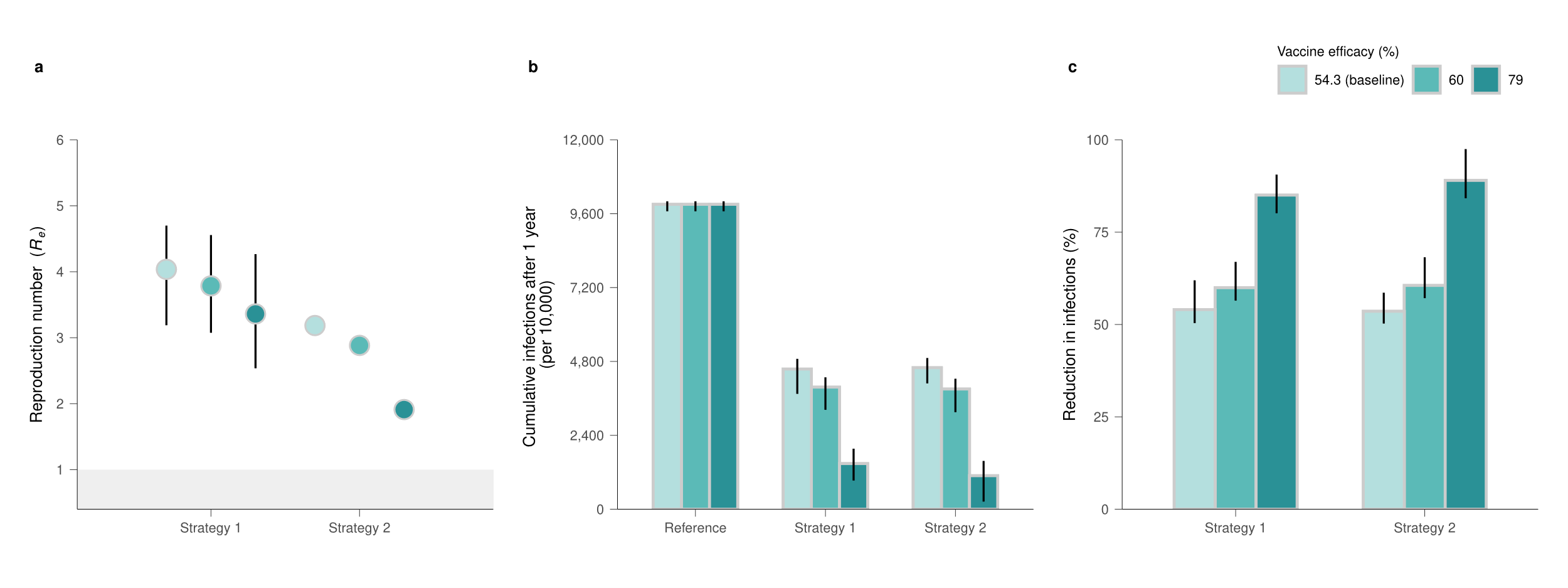


**Fig. S8 Sensitivity analysis on the maximum vaccine efficacy.** **a** Estimated effective reproduction number $R_{e}$ (mean and 95% CI) at the start of epidemic (December 1, 2021) for the two analyzed vaccination strategies. **b** Cumulative number of infections (mean and 95% CI) after 1 year for the *reference scenario* and the two analyzed vaccination strategies. **c** Reduction in infections (mean and 95% CI) due to vaccination with respect to the *reference scenario*.


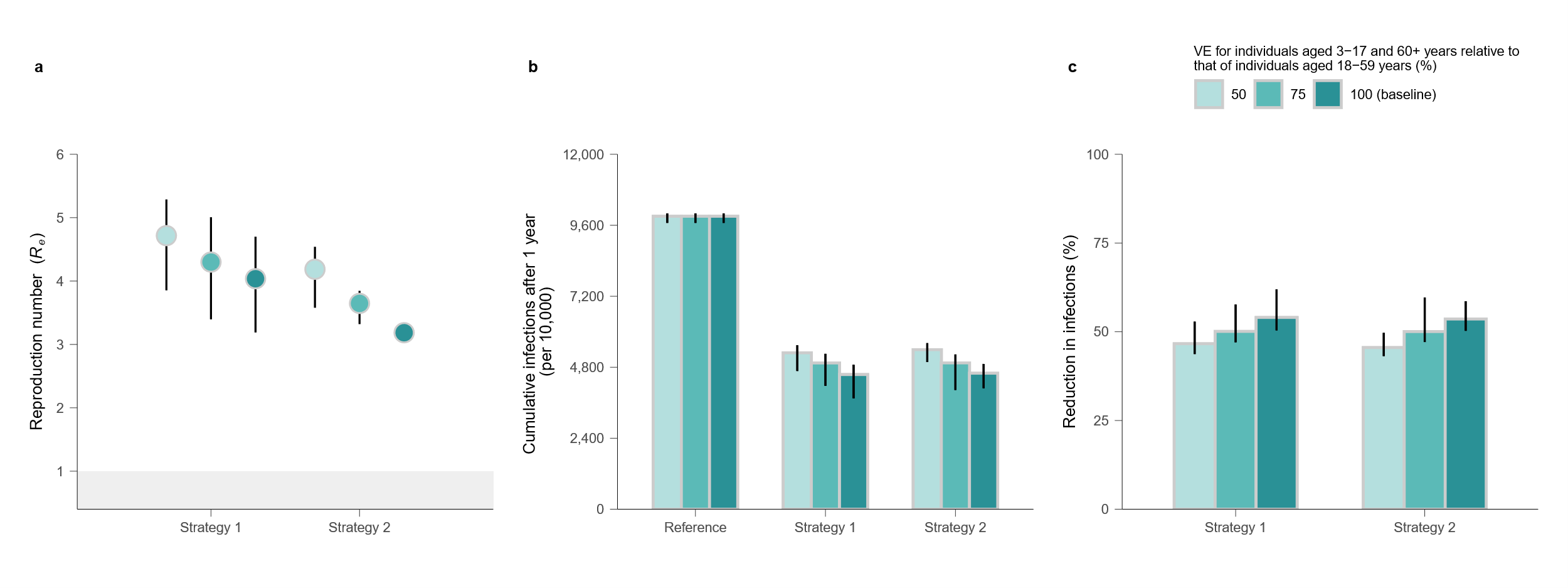
**Fig. S9 Sensitivity analysis on the relative vaccine efficacy for individuals aged 3-17 and 60+ years relative to that of individuals aged 18-59 years.** **a** Estimated reproduction number $R_{e}$ (mean and 95% CI) at the start of epidemic (December 1, 2021) for the two analyzed vaccination strategies. **b** Cumulative number of infections (mean and 95% CI) after 1 year for the *reference scenario* and the two analyzed vaccination strategies. **c** Reduction in the number of infections (mean and 95% CI) due to vaccination with respect to the *reference scenario*.

**
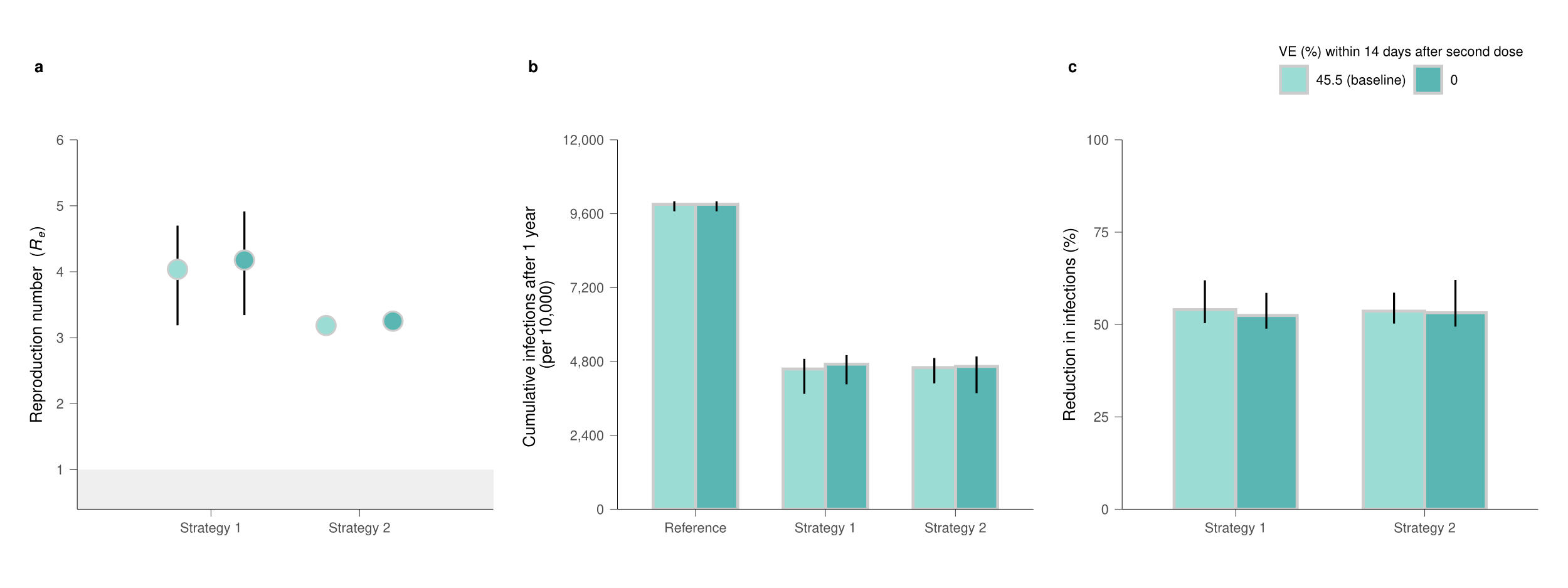
**

**Fig. S10 Sensitivity analysis on the vaccine efficacy within 14 days after second dose.** **a** Estimated effective reproduction number $R_{e}$ (mean and 95% CI) at the start of epidemic (December 1, 2021) for the two analyzed vaccination strategies. **b** Cumulative number of infections (mean and 95% CI) after 1 year for the *reference scenario* and the two analyzed vaccination strategies. **c** Reduction in the number of infections (mean and 95% CI) due to vaccination with respect to the *reference scenario*.

- 1. **Time interval between two doses**

In the main analysis, we considered a two-dose vaccine with a 21-day interval between two doses (Fig. 2). Here we proposed a sensitivity analysis on this time interval and it was set to be 14 days. The obtained results of two vaccination strategies are similar for two scenarios (Fig. S11).

**
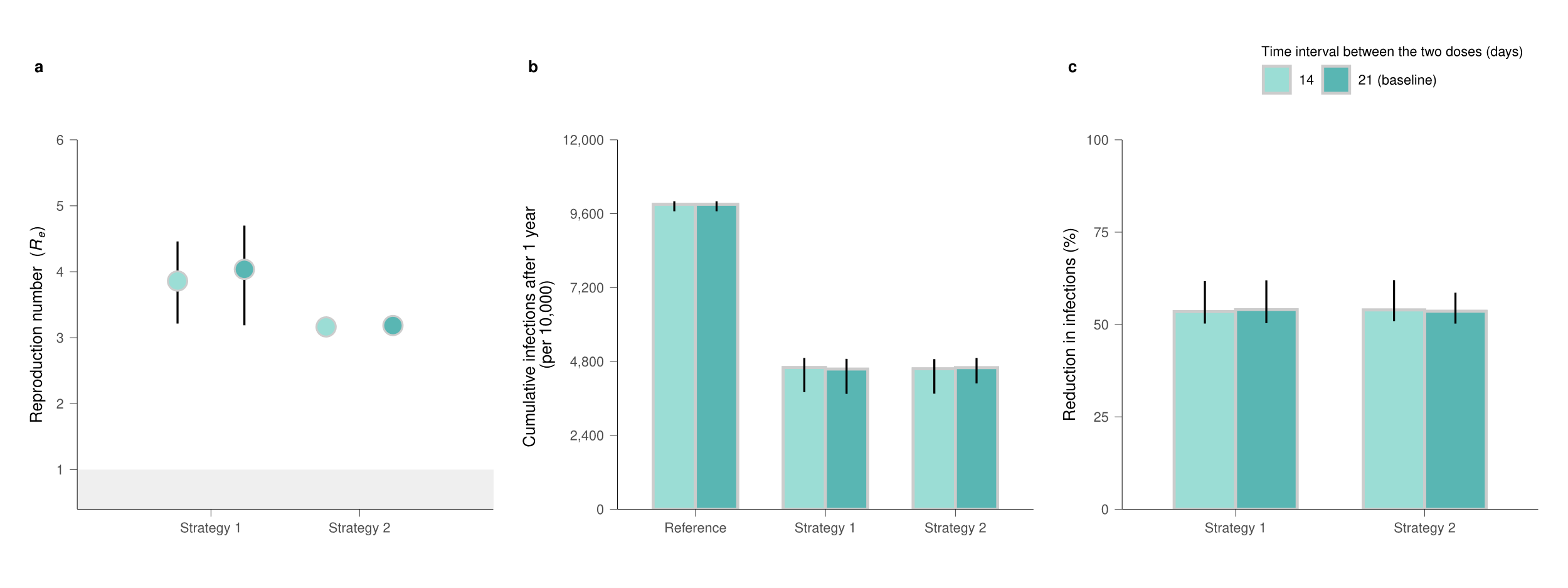
**

**Fig. S11 Sensitivity analysis on the time intervals between the two doses.** **a** Estimated effective reproduction number $R_{e}$ (mean and 95% CI) at the start of epidemic (December 1, 2021) for the two analyzed vaccination strategies. **b** Cumulative number of infections (mean and 95% CI) after 1 year for the *reference scenario* and the two analyzed vaccination strategies. **c** Reduction in the number of infections (mean and 95% CI) due to vaccination with respect to the *reference scenario*.

1. **Scenario 1: Delaying the start of the epidemic**

We tested to what extent the start of a new epidemic wave needs to be delayed (e.g., by keeping strict restriction for international travels) to allow the immunity to build up in the population, potentially reaching herd immunity levels in Fig. 3 a-c. Here we reported the impact of delaying the start of the epidemic on total vaccine coverage (Fig. S12 a-b) and daily incidence (Fig. S12 c-d).


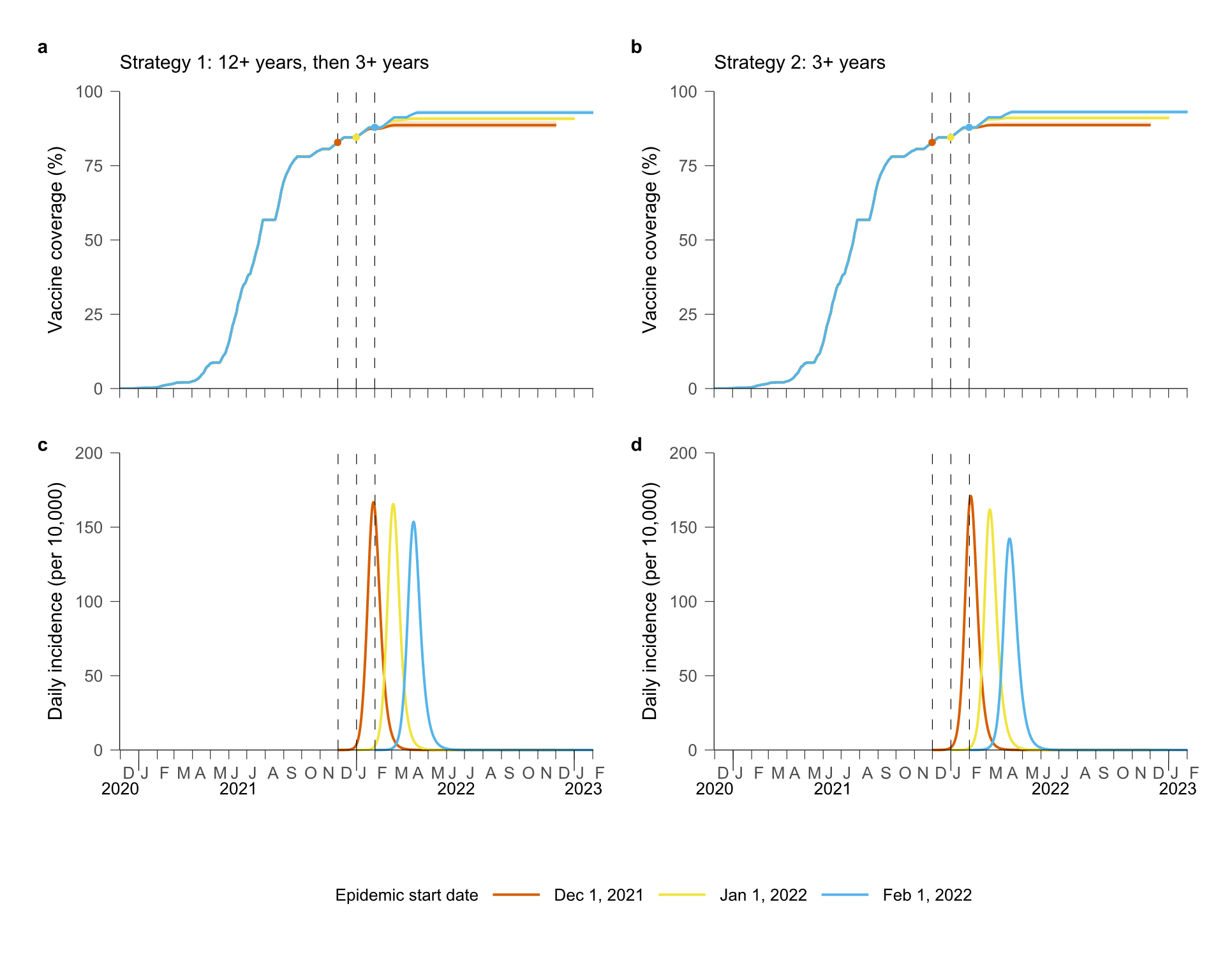


**Fig. S12 Impact of delaying the start of the epidemic on vaccine coverage and daily incidence** **a** Cumulative vaccination coverage of strategy 1. **b** As a, but for strategy 2. **c** Daily incidence of new infection for strategy 1. **d** As c, but for strategy 2.

1. **Scenario 2: Adopting NPIs in case of a new outbreak**

We investigated the synergetic effect of vaccination programs combined with NPIs of different intensity in Fig. 3 d-f. Here we showed the results of lager values of $R_{0}^{NPIs}$ (Fig. S13) and reported the impact of adopting NPIs on daily incidence (Fig. S14).


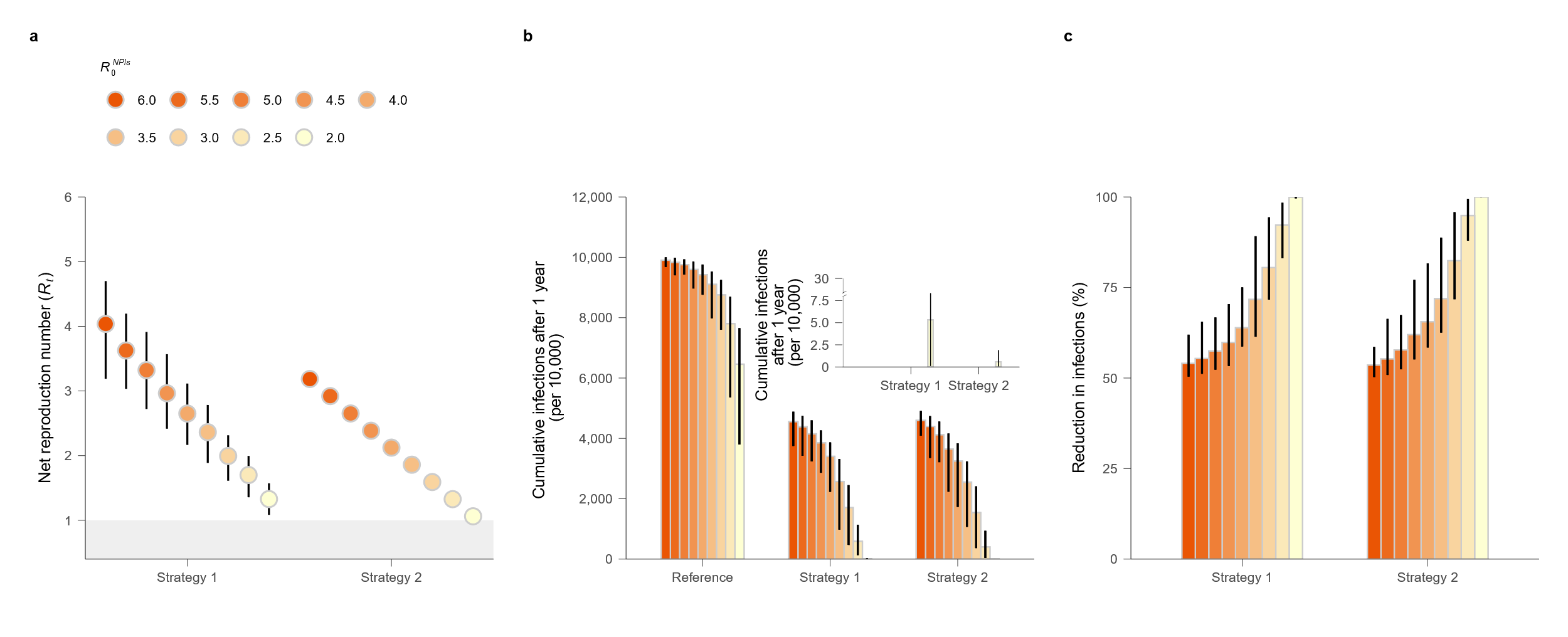


**Fig. S13 Impact of adopting NPIs in case of a new outbreak.** **a** Estimated net reproduction number (*R_t,_* mean and 95% CI) at the start of epidemic (December 1, 2021) adopting different intensity of NPIs, $R_{0}^{NPIs}$. **b** Cumulative number of infections per 10,000 individuals after 1 simulated year for *reference scenario* and two vaccination strategies (mean and 95% CI). **c** Reduction in infections (mean and 95% CI) with respect to the *reference scenario*.


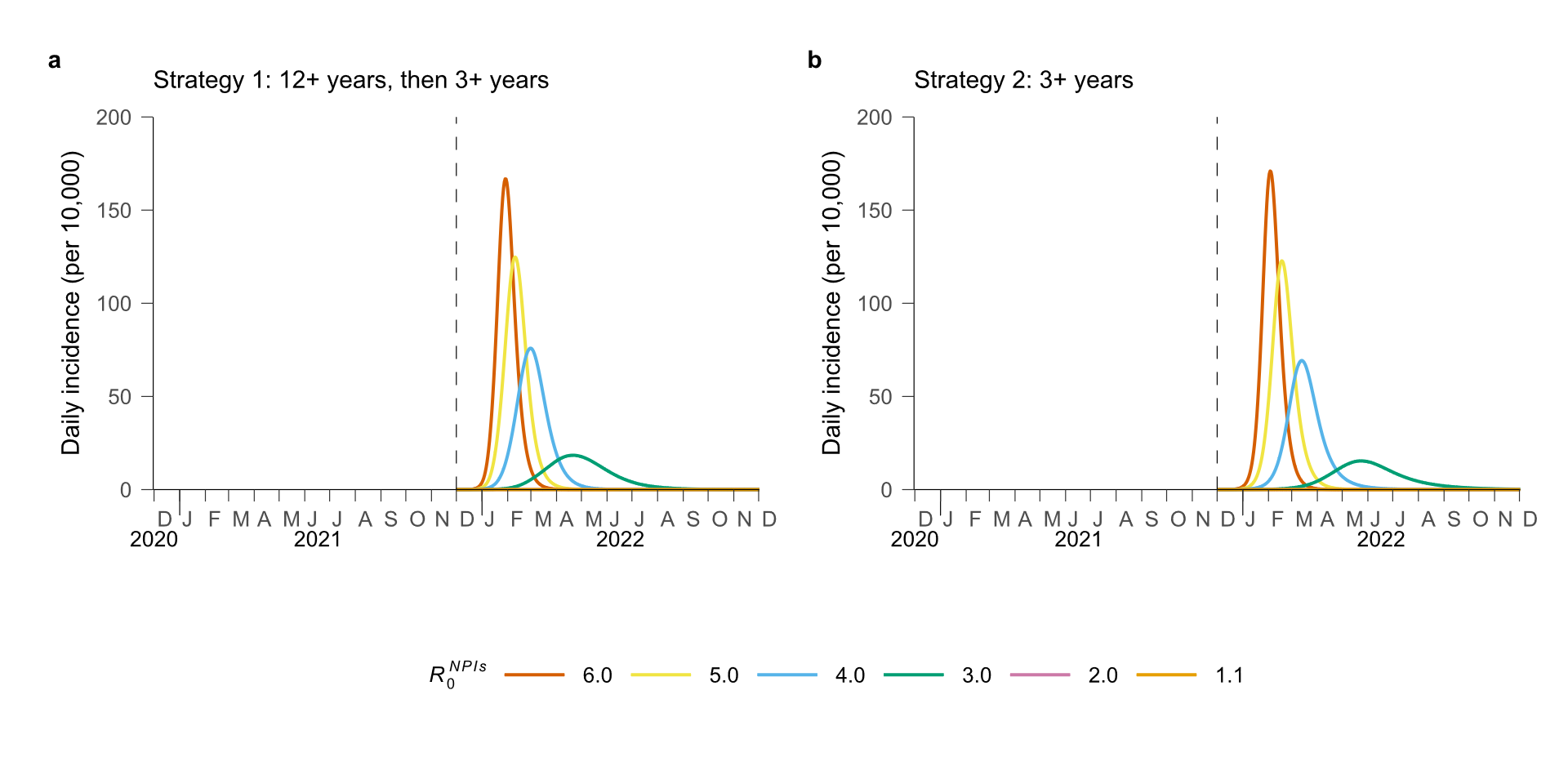


**Fig. S14 Impact of adopting NPIs in case of a new outbreak on daily incidence.** **a** Vaccine coverage of strategy 1. **b** Vaccine coverage of strategy 2.

2. **Scenario 3: Delaying the start of the epidemic and adopting NPIs**

We tested the combination of delaying the start of the epidemic and adopting NPIs on the effective reproduction number in Fig. 4. Here we reported the impact of them on infections (Fig. S15). In particular, the reduction in infections is more than 50% Besides, we performed other sensitivity analyses: exploring impact of the contact matrix considering an independently estimated contact matrix for the Chinese population [55] (Fig. S16). The obtained results show that $R_{e}$ is very consistent with those obtained in the main analysis (Fig. S17).


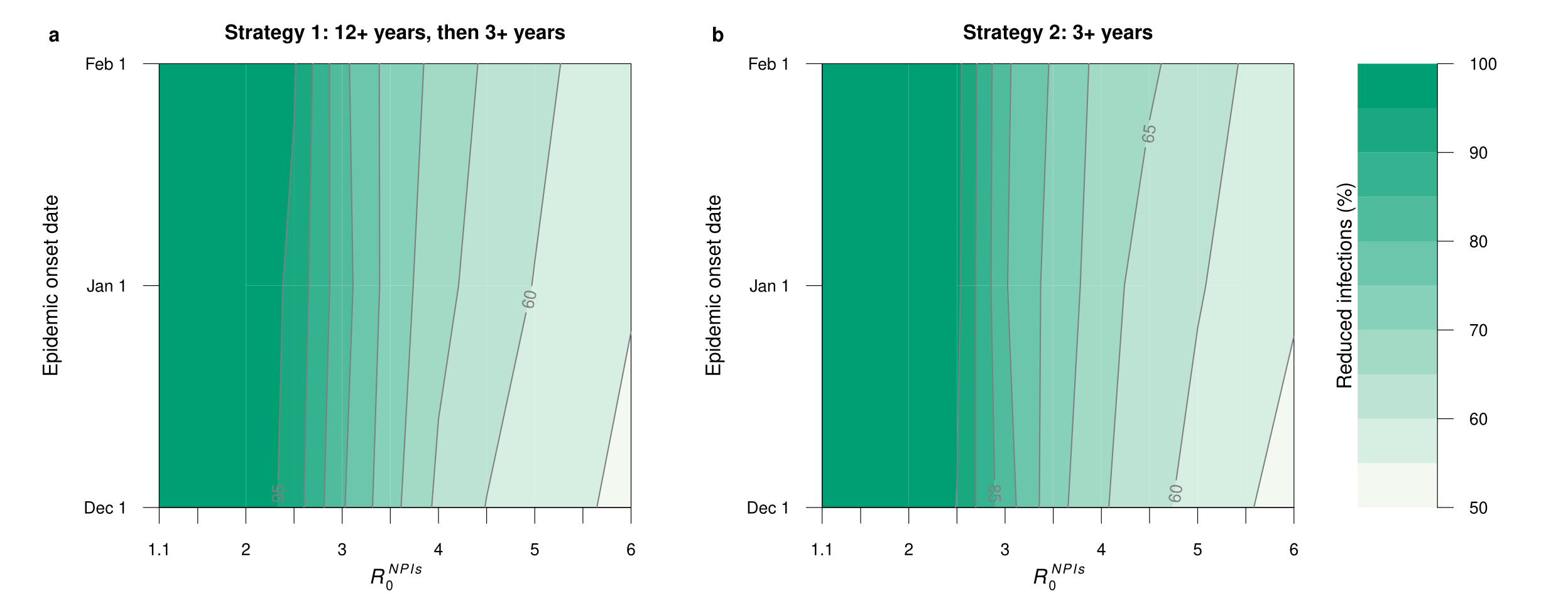


**Fig. S15 Impact of delaying the start of the epidemic and adopting NPIs on infections.** **a** Reduction in infections of strategy 1 with respect to the *reference scenario*. **b** As a, but for strategy 2.


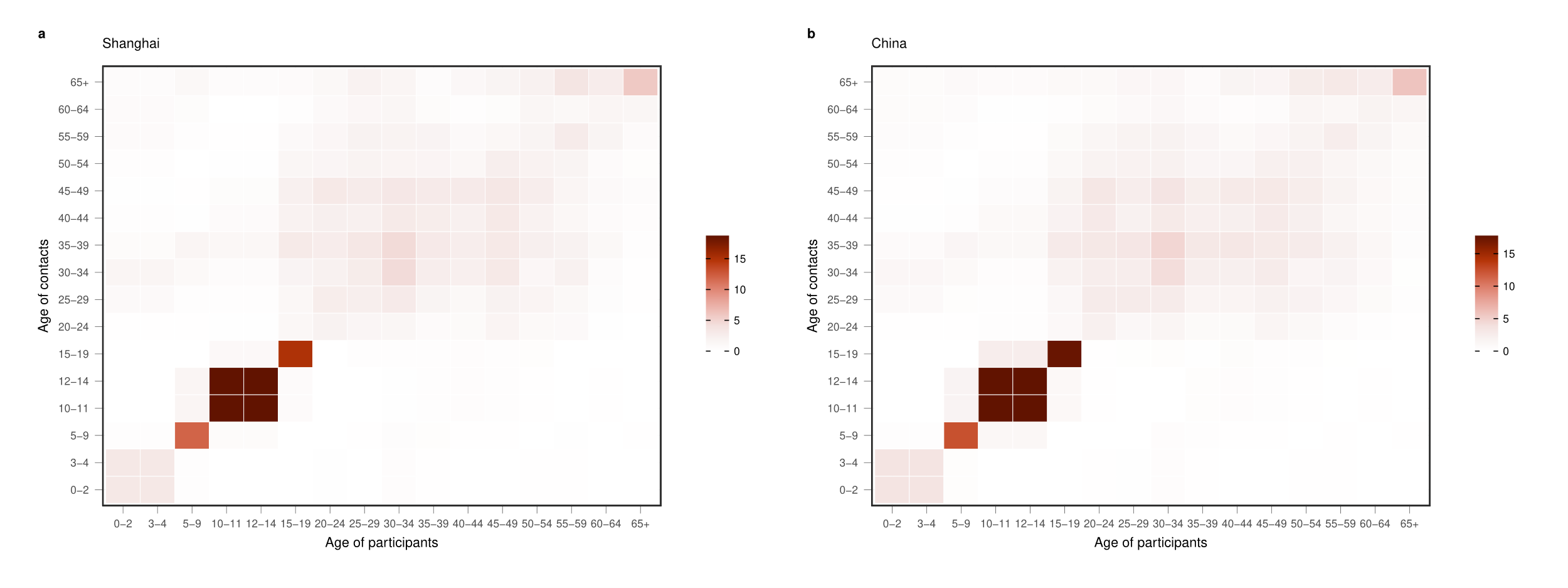


**Fig. S16 Comparison of contact matrix in Shanghai and China. a** Shanghai contact matrix. **b** Chinese contact matrix.


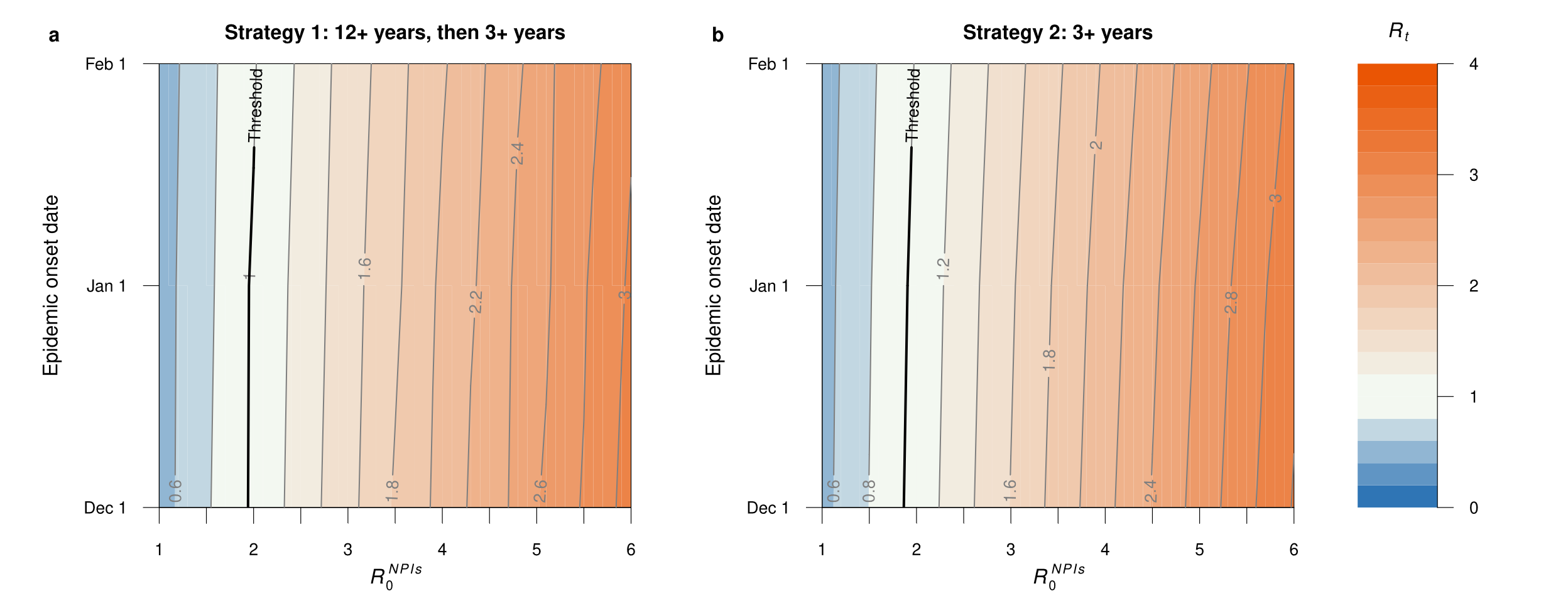


**Fig. S17 Impact of delaying the start of the epidemic start and adopting NPIs on estimated net reproduction number using China contact matrix.**  **a** Estimated net reproduction number (*R_t_*) as a function of *RNPIs 0*and epidemic start date for strategy 1. The bold line in black indicates the herd immunity threshold *R_t_* =1. **b** As a, but for strategy 2.

1. **Additional results about the herd immunity threshold**

We showed the relation between $R_{e}$ and vaccine coverage using an 95% efficacious vaccine (Fig. S18 a). The obtained results show that the herd immunity against Delta strain can be reached by using a higher efficacy and level of immunity needed to lead the effective reproduction number below the epidemic threshold is lower if vaccination is extended to individuals aged 3 years and older early on. Although the effective reproduction number is high when vaccine efficacy equals to 80%, while the infection attack rate is low (Fig. S18 b). Besides, we showed the impact of vaccine efficacy and vaccine coverage on estimated net reproduction number $R_{t}$ adopting different intensity of NPIs (Fig. S19). The obtained results show that the net reproduction number can be reduced below the unit in the presence of NPIs.


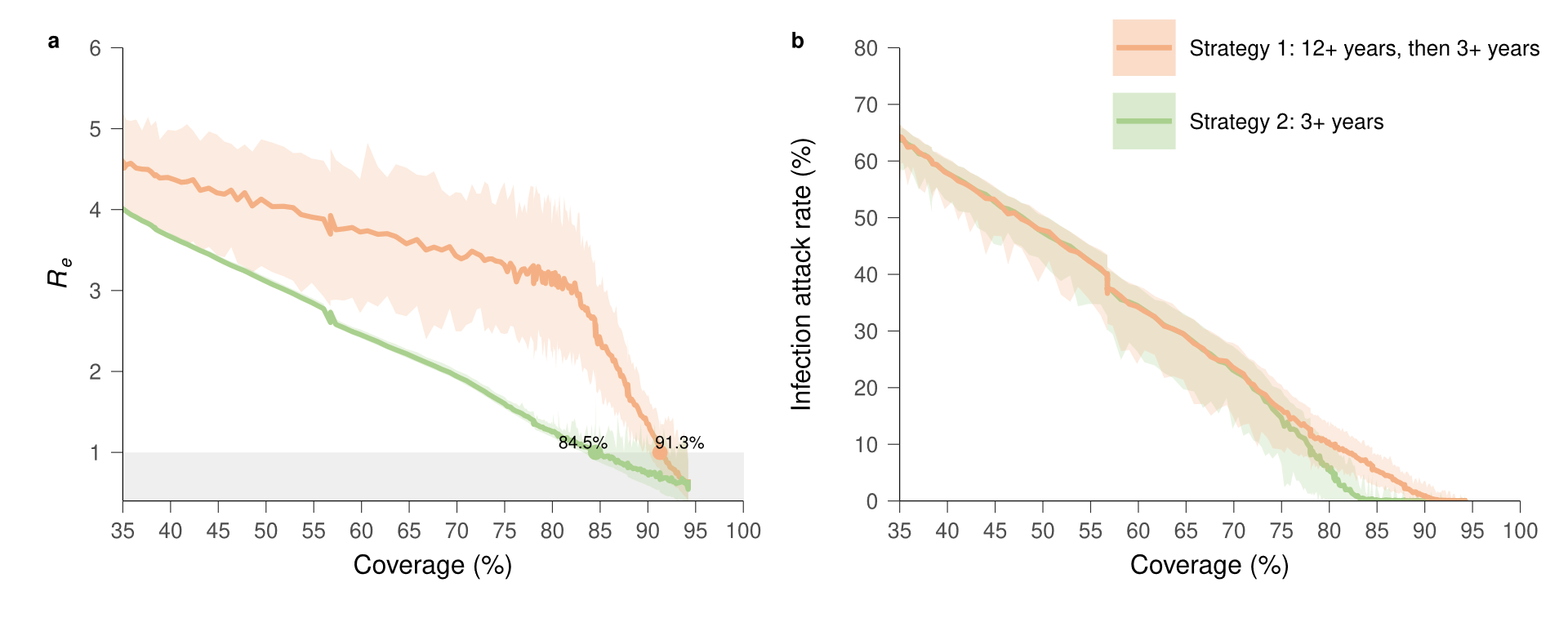


**Fig. S18 Effective reproduction number and infection attack rate under different vaccine coverage.** **a** Estimated effective reproduction number $R_{e}$ (mean and 95% CI) as a function of the vaccination coverage, assuming an 95% efficacious vaccine and no transmission. Colors refer to the three vaccination strategies. The shaded area in gray indicates the herd immunity threshold $R_{e}$=1. The numbers around the line indicate are the threshold of vaccine coverage. **b** The infection attack rate after 1 simulated year as a function of vaccine coverage with an 95% efficacious vaccine. The transmission is simulated with the initial vaccine coverage and there is no longer vaccination after the infection is seeded.


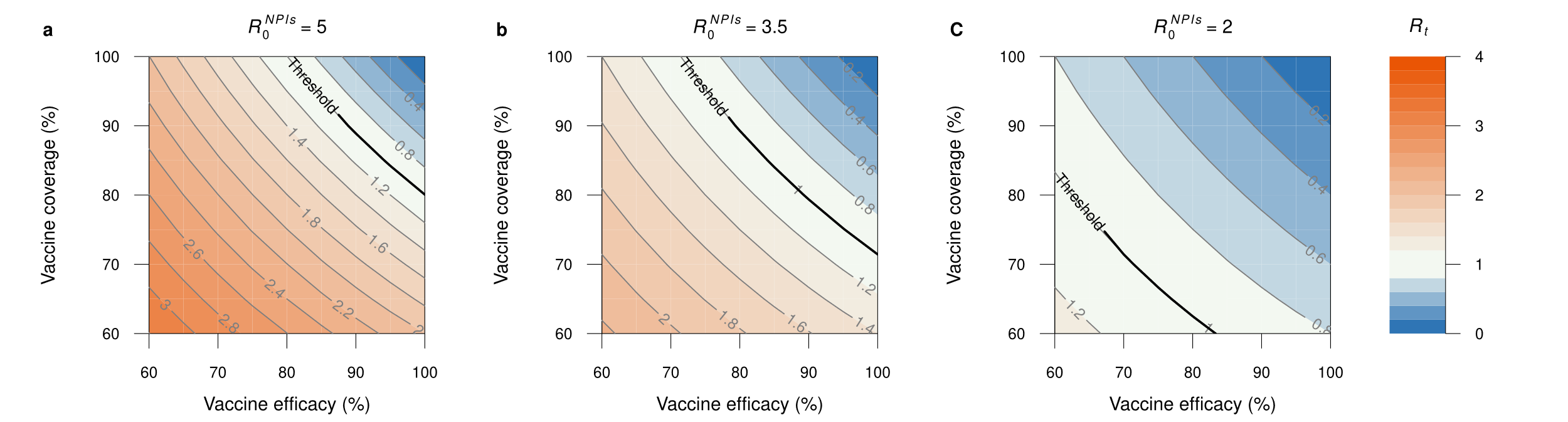


**Fig. S19 Impact of vaccine efficacy and vaccine coverage on estimated net reproduction number under different intensity of NPIs. a** $R_{0}^{NPIs}=5.0$. **b** $R_{0}^{NPIs}=3.5$. **c** $R_{0}^{NPIs}=2.0$.

1. **Model with no age structure**

We performed an analysis of considering a single age group thus the susceptibility, age-mixing pattern, and vaccine efficacy are homogenous (Fig. S20). We found that the cumulative infections after 1year is higher compared with the result of Fig. S5. And effective reproduction number of no age structure is apparently different from the result of strategy 1 using 16 age groups.


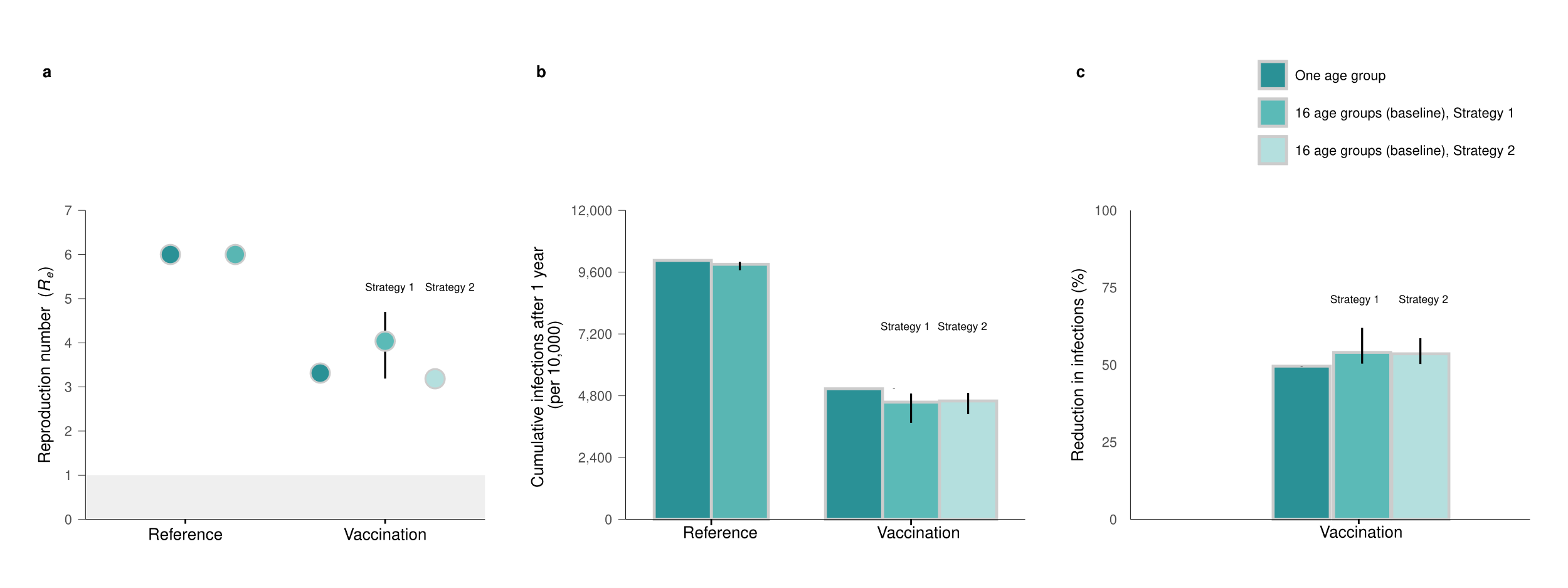


**Fig. S20 Results of model with no age structure.** **a** Estimated reproduction number $R_{e}$ (mean and 95% CI) at the start of epidemic (December 1, 2021) without/with vaccination program. **b** Cumulative number of infections (mean and 95% CI) after 1 year for the *reference scenario* and vaccination program. **c** Reduction in the number of infections (mean and 95% CI) due to vaccination program with respect to the *reference scenario*.

1. **Natural immunity and mRNA vaccine**

We proposed a sensitivity analysis where we simulated a scenario, where VE=79% [48] and the initial fraction of naturally immune population is set at 22% [64], finding remarkably different results both in terms of transmission potential and number of infections (Fig. S21).


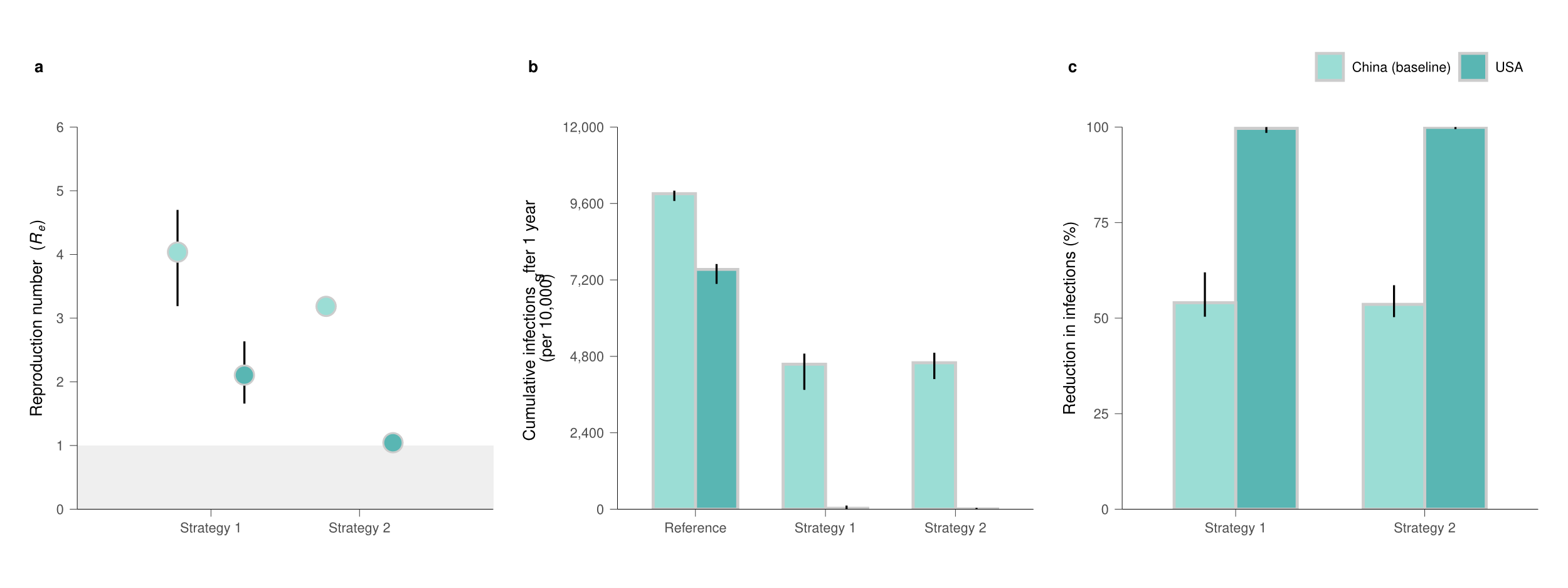


**Fig. S21 Comparison between China and a scenario with natural immunity and an mRNA vaccine.** **a** Estimated reproduction number $R_{e}$ (mean and 95% CI) at the start of epidemic (December 1, 2021) for the two analyzed vaccination strategies. **b** Cumulative number of infections (mean and 95% CI) after 1 year for the *reference scenario* and the two analyzed vaccination strategies. **C** Reduction in the number of infections (mean and 95% CI) due to vaccination with respect to the *reference scenario*.
